# Conformational gating governs nucleotide incorporation by a DNA-crosslinked polymerase

**DOI:** 10.1093/nar/gkag539

**Published:** 2026-06-02

**Authors:** Daniel Betancourt, Amit Gaur, Turner W Seay, Nikita Zalenski, Zucai Suo

**Affiliations:** Department of Biomedical Sciences, College of Medicine, Florida State University, Tallahassee, FL 32306, United States; Department of Biomedical Sciences, College of Medicine, Florida State University, Tallahassee, FL 32306, United States; Department of Biomedical Sciences, College of Medicine, Florida State University, Tallahassee, FL 32306, United States; Department of Biomedical Sciences, College of Medicine, Florida State University, Tallahassee, FL 32306, United States; Department of Biomedical Sciences, College of Medicine, Florida State University, Tallahassee, FL 32306, United States

## Abstract

Base excision repair is a major pathway that repairs single-base DNA damage. We recently demonstrated that human DNA polymerase β (hPolβ) fills single-nucleotide gaps after Schiff base formation but before β-elimination, implying that its dRP lyase domain remains covalently crosslinked to DNA during gap-filling synthesis. Because uncrosslinked Polβ dissociates rapidly from DNA (∼3 s^−1^), mechanistic investigation has been challenging. To elucidate the kinetic mechanism of correct incorporation by DNA-crosslinked hPolβ, we generated a catalytically active crosslinked hPolβ‒DNA complex and performed pre-steady-state kinetic, thermodynamic, and structural analyses. Sulfur elemental effects of 3.7 ± 0.4 and 24 ± 4 for correct and incorrect nucleotide incorporation, respectively, suggest the chemical step is rate-limiting for incorrect, but not for correct, nucleotide incorporation. Pulse-chase and pulse-quench assays revealed a 33% difference in reaction amplitude, establishing the existence of a ternary intermediate preceding the chemical step. Eyring analysis identified a high activation free energy barrier, while the lack of viscosity dependence rules out large domain motions, indicating that the rate-limiting pre-chemical step involves local active-site rearrangements. Together with structurally characterized intermediates, these findings establish the first minimal kinetic mechanism for correct nucleotide incorporation by a DNA-crosslinked polymerase and identify local active-site rearrangements as the rate-limiting step.

## Graphical Abstract

**Graphical Abstract:**
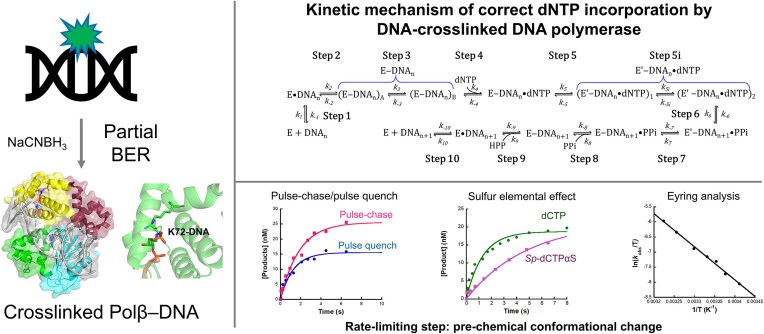

## Introduction

DNA base excision repair (BER) is a vital cellular pathway responsible for repairing over 10,000 single-base lesions per cell per day [1]. These lesions arise from diverse sources, both endogenous (e.g. oxidative damage, deamination, alkylation, and depurination/depyrimidination) and exogenous [1–5]. Uracil, for instance, can arise from either cytosine deamination or dUMP misincorporation during DNA synthesis [6]. Uracil repair in mammals involves four enzymes: uracil–DNA glycosylase (UDG) removes the uracil, AP endonuclease (APE1) cleaves the DNA backbone, DNA polymerase β (Polβ) fills the gap, and DNA ligase III/XRCC1 or DNA ligase I seals the nick (Supplementary Fig. S1) [7]. BER dysfunction is found in various human diseases, including cancer, premature aging, neurodegenerative disorders, and inherited DNA repair deficiencies [8–10]. Notably, aberrant human Polβ (hPolβ) variants can induce genome instability and carcinogenesis [11], and hPolβ mutations are found in ∼30% of all cancers and up to 40% of colorectal tumors [12]. Therefore, a thorough understanding of hPolβ function is essential for developing new therapies for numerous debilitating human diseases.

**Figure 1.**
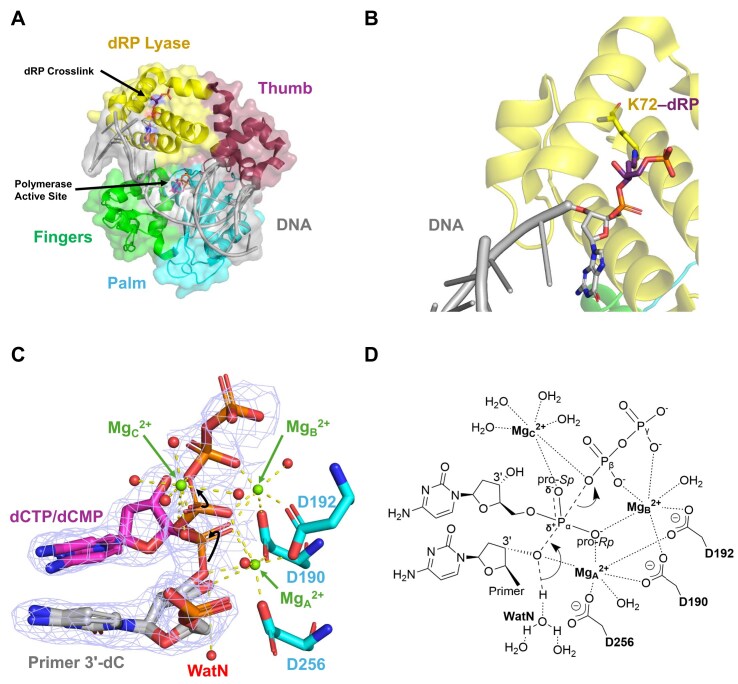
Crystal structures of DNA-crosslinked hPolβ and a proposed transition state. (**A**) Crystal structure of the crosslinked complex hPolβ‒DNA^dRP^ (PDB: 7RBG). The dRP lyase domain is in yellow while thumb subdomain is in maroon, palm is cyan, fingers is green, and DNA is gray. The arrows point to the crosslinked site and the polymerase active site. (**B**) Zoomed view of crosslinked K72‒dRP (PDB: 7RBG). (**C**) Zoomed reaction-state structure (PDB: 7RBK). Electron density *F_o_–F_c_* omit map (light blue; 3σ) following 40 s of Ca^2+^ to Mg^2+^ exchange *in crystallo* shows the upstream primer 3′-dC, 30% remaining dCTP, and 70% incorporated dCMP. The simultaneous phosphodiester bond formation and Pα‒O bond breakage, along with the steric inversion of the Pα geometry, indicate an S_N_2 reaction between the 3′-dC and dCTP. Spheres represent Mg^2+^ (green) and water (red). (**D**) Proposed transition state for phosphodiester bond formation based on the reaction-state structure in panel (C). The chemical bonds undergoing cleavage and formation during the S_N_2 reaction are represented by dashed lines while the hydrogen bonds and the coordination bonds to the three Mg^2+^ ions are denoted by dotted lines.

Polβ, a ubiquitously expressed eukaryotic DNA polymerase, is composed of a single polypeptide chain comprising an N-terminal 8-kDa dRP lyase domain and a 31-kDa C-terminal polymerase domain (Fig. 1A) [13]. The C-terminal domain is further subdivided into palm, thumb, and finger subdomains, each serving functions in substrate recognition and binding, and conformational dynamics for nucleotidyl transferase catalysis (Fig. 1A) [13]. Uncrosslinked Polβ exhibits nucleotide incorporation rates that are typically 10–100-fold lower than those of replicative polymerases and displays weaker DNA binding (>100-fold) along with a pronounced preference for gapped DNA substrates, consistent with its role in base excision repair [14–18]. Despite lacking a dedicated proofreading activity, DNA-crosslinked and uncrosslinked Polβ exhibits moderate error frequencies of 10^−5^ during gap-filling DNA synthesis *in vitro* [7].

**Figure 2.**
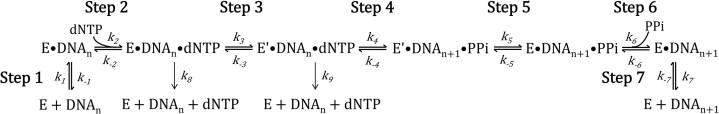
Minimal kinetic mechanism of nucleotide incorporation into a single-nucleotide gapped DNA substrate catalyzed by uncrosslinked hPolβ. E●DNA_n_ and E●DNA_n+1_ refer to the binary complexes of uncrosslinked hPolβ with DNA_n_ and DNA_n+1_, respectively.

Uncrosslinked Polβ has served as a model enzyme for mechanistic studies due to its robust stability, relatively small size, and lack of accessory subunits [19–24]. These studies demonstrate that uncrosslinked Polβ shares a minimal kinetic mechanism with other uncrosslinked DNA polymerases, reverse transcriptases, and RNA polymerases (Fig. 2) [22, 24]. This mechanism involves orderly substrate binding to the polymerase (E), beginning with DNA_n_ binding (Step 1) to form the binary complex E●DNA_n_, followed by nucleotide association (Step 2) to form the ground-state ternary complex E●DNA_n_●dNTP. This ternary complex undergoes a protein conformational change (Step 3) to form a tight ternary complex E′●DNA_n_●dNTP with precise alignment of the templating nucleotide, an incoming nucleotide, and divalent metal ions within the polymerase active site [19]. Phosphodiester bond formation between the incoming dNTP and the DNA primer results in forming the E′●DNA_n+1_●PPi complex (Step 4) with elongated DNA_n+1_ and pyrophosphate (PPi). A reverse protein conformational change (Step 5) returns the polymerase to its open conformation in E●DNA_n+1_●PPi, enabling PPi dissociation (Step 6) and subsequent release of DNA_n+1_ (Step 7).

Despite this well-defined framework, the identity of the rate-limiting step for correct nucleotide incorporation by uncrosslinked Polβ has remained contested [15, 19–22, 25, 26]. Early crystallographic and pre-steady-state analyses established an induced-fit mechanism in which nucleotide binding promotes finger-domain closure and catalytic metal assembly [16, 27–29]. Stopped-flow 2-aminopurine studies indicated that this conformational closure is rapid, suggesting that later steps dominate the observed rate [30–33]. Sulfur elemental-effect measurements for correct incorporation are modest [15], arguing against the chemical step being the sole rate-limiting step. In contrast, Brønsted leaving-group analyses detect measurable chemical sensitivity [34, 35], implying partial chemical character in the observed transition and suggesting coupling between pre-chemical activation and early chemical events. Ensemble and single-molecule FRET experiments further revealed additional intermediates and a metal-binding phase not resolved by earlier fluorescence assays [14, 36–38], indicating that active site organization involves more complex dynamics than a simple two-state, open-closed transition. Recently, we were able to confirm the presence of a kinetically dominant pre-chemistry species via pulse-quench and pulse-chase assays, supporting a rate-limiting pre-chemical conformational transition during correct nucleotide incorporation by uncrosslinked hPolβ [24]. Complementary computational analyses describe a “pre-chemistry” activation landscape in which stochastic active-site rearrangement and metal coordination precede a comparatively low-barrier phosphoryl transfer step [39–41]. However, other experimental and computational studies have reported alternative interpretations, including contributions from the chemical step or partially coupled transitions [34, 35]. Collectively, these studies highlight the complexity of the system and indicate that, although a pre-chemical conformational transition plays a significant role, the precise identity of the rate-limiting step for uncrosslinked hPolβ remains contested.

A major challenge in resolving this question arises from the transient nature of intermediate states and the rapid dissociation of DNA from uncrosslinked hPolβ, which limits direct kinetic interrogation of enzyme-bound species. Prior mechanistic studies of Polβ have relied on non-physiological DNA substrates, such as ungapped DNA, gapped DNA, or gapped DNA containing a 5′-phosphate or both a 5′-phosphate and an unnatural deoxyribose (dRP) mimic, 5′-tetrahydrofuran, on the downstream primer (Supplementary Fig. S2A–D) [20–22]. While these approaches have enabled detailed mechanistic analyses, they preclude formation of the native covalent intermediate and therefore limit direct investigation of enzyme-bound states under physiologically relevant conditions. In contrast, our mechanistic and structural work used a physiologically relevant DNA substrate containing an *in situ* generated abasic site (Supplementary Fig. S2E), revealing a significant revision to the hPolβ-catalyzed reaction sequence during BER: gap-filling DNA synthesis by the polymerase activity occurs after Schiff base formation but before β-elimination by the dRP lyase activity (Supplementary Fig. S1) [7]. These findings indicate that *in vivo*, gap-filling DNA synthesis proceeds while hPolβ remains covalently linked to DNA (Fig. 1B). This covalent intermediate enables kinetic interrogation of enzyme-bound species that are otherwise transient in studies of uncrosslinked Polβ [19, 22, 42] and provides an opportunity to more clearly define the rate-limiting step. Because the DNA-crosslinked Polβ complex differs structurally from the binary complex of uncrosslinked Polβ [7], crosslinking may alter conformational dynamics and modulate kinetic partitioning during catalysis. We therefore performed a comprehensive pre-steady-state kinetic and structural analysis of single-nucleotide gap filling synthesis by DNA-crosslinked hPolβ to define its minimal kinetic mechanism under physiologically relevant conditions.

## Materials and methods

### Reagents

All chemicals and reagents used were of Reagent Grade purity or higher. Radiolabeled nucleotides [γ-^32^P]ATP and [α-^32^P]dCTP were purchased from Revvity Health Sciences, unlabeled dNTPs from Fisher Scientific, and *Sp* isomers of dNTP α-thiol analogs (>95% *Sp*) from Trilink Biotechnologies. All DNA oligonucleotides were purchased from Integrated DNA Technologies and purified through 20% urea polyacrylamide gel electrophoresis (PAGE). Bio-Spin 6 columns were purchased from Bio-Rad Laboratories. T4 polynucleotide kinase (UniProt accession ID: P06855) used to label DNA substrate was expressed and purified through column chromatography following published procedures [43]. Both hPolβ (UniProt accession ID: P06746) and human APE1 (UniProt accession ID: P27695) were individually expressed and purified as described previously [7].

### Preparation of crosslinked hPolβ‒DNA^dRP^ complex and *in situ* DNA substrate labeling

**Figure 3.**
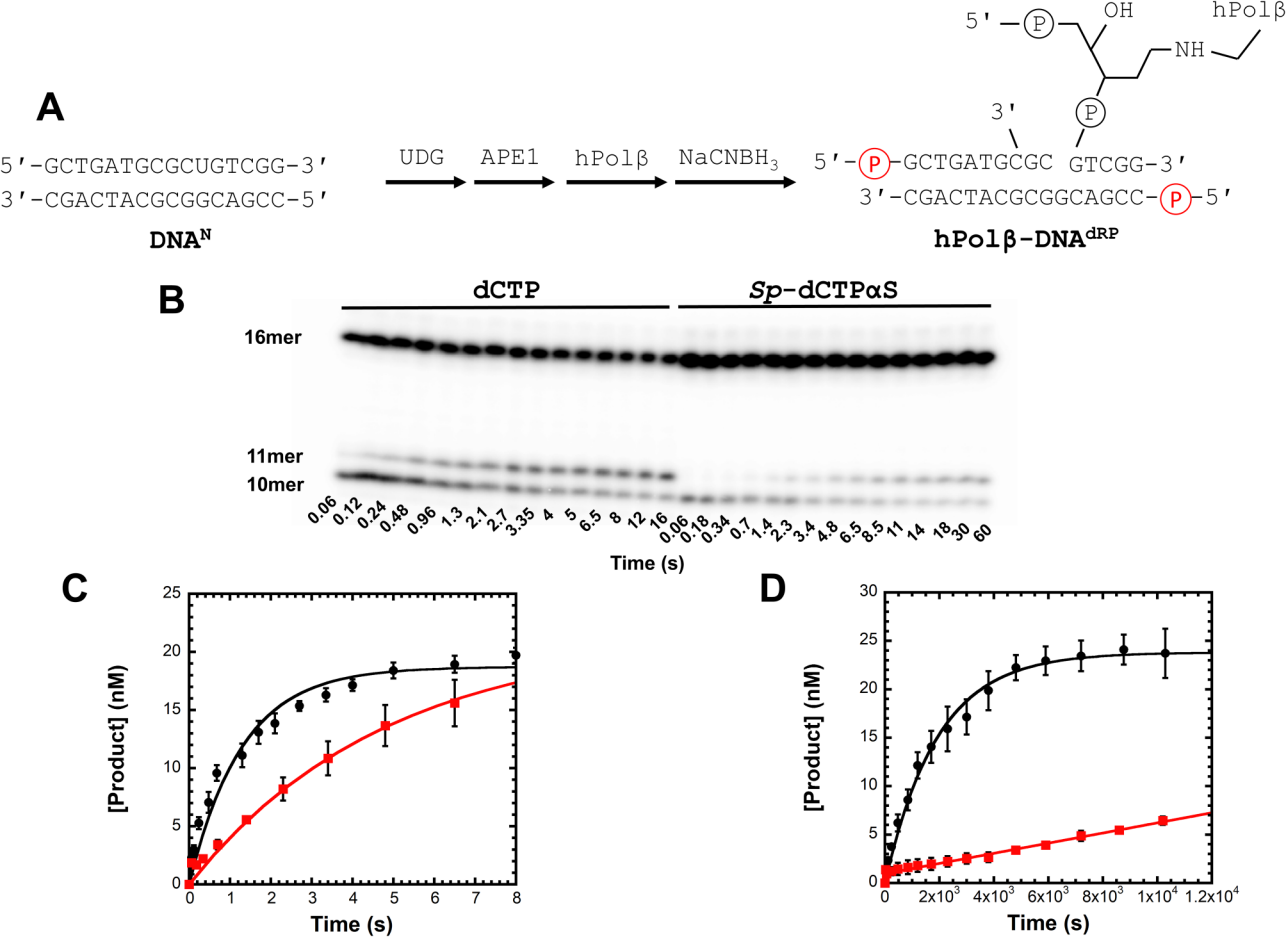
Sulfur elemental effect of nucleotide incorporation by DNA-crosslinked hPolβ. (**A**) The DNA^N^ substrate containing a deoxyuridine was sequentially processed by UDG, APE1, and hPolβ, followed by reduction with NaCNBH_3_ to form the crosslinked complex hPolβ‒DNA^dRP^. The purified hPolβ‒DNA^dRP^ complex was [^32^P]-labeled *in situ* which resulted in radiolabeling of the 5′-termini of the upstream 10mer primer and the 16mer template. The downstream 5mer, which was covalently crosslinked to hPolβ, was not radiolabeled. (**B**) Representative denaturing gel showing time-dependent incorporation of 25 µM dCTP (left) and 25 µM *Sp* -dCTPαS (right) by hPolβ‒DNA^dRP^. (**C**) A solution of hPolβ‒DNA^dRP^ (30 nM) was rapidly mixed with 25 µM dCTP (●) or 25 µM *Sp*-dCTPαS (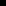) for varying times before quenching. The resulting time courses from three independent experiments were fit individually to Equation 1 (Materials and methods). Kinetic parameters represent mean ± SD (*n* = 3). The reaction amplitude (*A*) was 18.8 ± 0.6 nM and a *k*_obs_ of 0.77 ± 0.08 s^−1^ for dCTP incorporation, and *A* of 21.5 ± 2.8 nM and a *k*_obs_ of 0.21 ± 0.01 s^−1^ for *Sp*-dCTPαS incorporation. (**D**) The same reactions were performed with 400 µM dGTP (●) or 400 µM *Sp*-dGTPαS (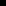). The dGTP misincorporation time course was fitted to Equation 1, giving an *A* of 23.8 ± 0.4 nM and a *k*_obs_ of 5.2 ± 0.3 × 10^−4^ s^−1^. The *Sp*-dGTPαS misincorporation time course was linear and was fit by linear regression to yield a slope of 5.2 ± 0.2 × 10^−4^ nM/s. Curves shown represent the average of three independent experiments; error bars denote the standard error of the mean at each time point.

Preparation of the crosslinked hPolβ‒DNA^dRP^ complex proceeded as described previously [7]. Briefly, complementary DNA oligonucleotides 5′-GCTGATGCGCUGTCGG-3′ (containing a 2′-deoxyuridine (dU) residue) and 5′-CCGACGGCGCATCAGC-3′ were annealed in 20 mM Tris–HCl (pH 7.9) by heating the solution to 95°C for 5 min, followed by gradual cooling to room temperature and then to –20°C for at least 3 h [44]. The resulting double-stranded DNA^N^ (Fig. 3A) was incubated with uracil DNA glycosylase (UDG; UniProt accession ID: P12295) from New England Biolabs and APE1 at 37°C in crosslinking buffer [10 mM HEPES (pH 7.5), 50 mM KCl, 5 mM MgCl_2_, and 1 mM dithiothreitol (DTT)] for 25 min (Fig. 3A). The reaction was then placed on ice and purified hPolβ was crosslinked with the processed DNA^dRP^ substrate (1:1.5 molar ratio) on ice for 30 min in the presence of 350 mM NaCNBH_3_ (Supplementary Fig. S3). Free DNA^dRP^, uncrosslinked hPolβ, UDG, APE1, and unreacted NaCNBH_3_ were removed by gel filtration. Finally, the isolated hPolβ‒DNA^dRP^ complex was dialyzed into storage buffer (50 mM Tris‒HCl, pH 7.5 at 4°C, 100 mM NaCl, 50% glycerol, and 0.1% 2-mercaptoethanol), concentrated, aliquoted, and stored at –80°C for subsequent experiments. hPolβ‒DNA^dRP^ was 5′-radiolabeled by incubation with T4 kinase and [γ-^32^P]ATP in T4 kinase buffer (70 mM Tris‒HCl, pH 7.5 at 37°C, 10 mM MgCl_2_, and 5 mM DTT) for 30 min at room temperature.

### DNA substrate labeling and annealing

For experiments with uncrosslinked hPolβ, a single-nucleotide gapped DNA substrate DNA^P^ (Supplementary Fig. S2C) was prepared by annealing oligonucleotides 16mer (5′-CCGACGGCGCATCAGC-3′), 10mer (5′-GCTGATGCGC-3′), and 5mer (5′-GTCGG-3′) in a 1:1.2:1.15 molar ratio, following the annealing protocol as described above. The 5mer was 5′-phosphorylated but not radiolabeled, while the upstream primer 10mer was 5′-radiolabeled by incubation with T4 polynucleotide kinase and [γ-^32^P]ATP for 3 h at 37°C. Following heat inactivation at 95°C for 5 min, the inactivated kinase and unreacted [γ-^32^P]ATP were removed by centrifugation through a Bio-Spin 6 column [45].

### Pre-steady-state kinetic assays

Pre-steady-state kinetic experiments were performed at 37°C immediately following labeling in optimized hPolβ reaction buffer [50 mM Tris–HCl (pH 7.8 at 37°C), 5 mM MgCl_2_, 50 mM NaCl, 5 mM DTT, 10% glycerol, 0.1 mg/ml BSA, and 0.1 mM EDTA to chelate any contaminating divalent metal ions] unless otherwise stated. Reactions were rapidly mixed and quenched using an RFQ-3 Rapid-Chemical Quench (KinTek Corporation) [46, 47] as described previously [48–51]. A computer-controlled motor rapidly mixed equal volumes (15 µl) of reacting solutions: one containing the pre-formed enzyme–DNA complex and the other containing dNTP (and MgCl₂ where indicated), followed by rapid mixing with the quenching solution (80 µl) at the designated reaction time. Reactions were quenched with either EDTA (0.37 M) or HCl (1 M) as specified in the experiment [52, 53]. Experiments with long reaction times were manually performed and quenched. All concentrations are reported as final values after mixing.

### Viscosity dependence of nucleotide incorporation

To examine the effect of solution viscosity on the rate of nucleotide incorporation, pre-steady-state kinetic assays were performed with glycerol concentrations ranging from 0% to 30% (v/v). Glycerol was added to both enzyme and nucleotide solutions. All other reaction conditions were identical to those described above.

### Temperature-dependence assays

To determine the temperature dependence of nucleotide incorporation, pre-steady-state kinetic experiments were performed between 20 and 37°C. The buffer pH was maintained constant across all temperatures. All other reaction conditions were unchanged.

### Measurement of the elemental effect on nucleotide incorporation

To assess the elemental effect, a preincubated solution containing radiolabeled hPolβ‒DNA^dRP^ complex (30 nM) was rapidly mixed with a solution containing either dCTP or *Sp*-dCTPαS (25 µM). The reaction was allowed to proceed for varying time intervals and then quenched with 0.37 M EDTA. DNA polymerases are known to exhibit stereoselectivity such that nearly pure (≥95%) *Sp* isomer was used [54]. The elemental effect on mismatched nucleotide incorporation was determined under identical assay conditions, except that dGTP or *Sp-*dGTPαS (400 µM) was used instead.

### Pulse-quench and pulse-chase assay

A preincubated solution containing unlabeled hPolβ‒DNA^dRP^ (30 nM) and Mg^2+^ was rapidly mixed with a solution containing [α-^32^P]dCTP (1.5 µM). In the pulse-quench assay, the reaction was immediately terminated with 1 M HCl at various times. For the pulse-chase assay, reactions were chased with a large excess of unlabeled dCTP (1.5 mM) for 20 s before quenching with 1 M HCl. After quenching, both pulse-quench and pulse-chase reactions were subjected to chloroform to separate and denature the enzyme and then immediately neutralized with 1 M NaOH prior to analysis.

### Product analysis

Reactions were denatured by heating to 95°C for 5 min in formamide loading buffer (40% [v/v] formamide, 1× TBE, 10 mM EDTA, 0.025% [w/v] bromophenol blue, and 0.025% [w/v] xylene cyanol) and subsequently products were resolved by denaturing Urea‒PAGE (20% acrylamide, 1% bis-acrylamide, and 8 M urea). Radiolabeled products were visualized and quantified with a Typhoon Biomolecular Imager (Cytiva). Gel images were analyzed with ImageQuant (Cytiva), and background-corrected intensities were used to determine the amount of product formed. To establish the concentration of products in pulse-quench and pulse-chase experiments, a standard curve was built by blotting a series of [α-^32^P]dCTP dilutions onto thin-layer chromatography plates and then imaging and analyzing as above.

### Data analysis

Data were analyzed by nonlinear regression using KeleidaGraph software (Synergy Software). The time course of product formation was fit to Equation 1:


(1) \begin{eqnarray*}
\left[ {\textit{product}} \right] = A\left[ {1 - {{e}^{ - {\mathrm{\ }}{{k}_{obs}}t}}} \right]
\end{eqnarray*}

Where *A* denotes a reaction amplitude, *k*_obs_ is an observed single-turnover rate constant, and *t* is the reaction time.

Rate constants determined at various temperatures were fit to the Eyring equation:


(2) \begin{eqnarray*}
k = \frac{{\kappa {{K}_B}T}}{h}{{e}^{\frac{{{\mathrm{\Delta }}{{S}^\ddagger }}}{R}}}{{e}^{ - \frac{{\vartriangle {{H}^\ddagger }}}{{RT}}}}
\end{eqnarray*}

Where $\kappa $ is the transmission coefficient, ${{K}_B}$ is the Boltzmann constant, $T$ is the temperature in kelvins, $h$ is the Planck constant, $R$ is the ideal gas constant, ${\mathrm{\Delta }}{{S}^\ddagger }$ is the activation entropy, and $\vartriangle {{H}^\ddagger }$ is the activation enthalpy.

### Crystallization and structure determination of the hPolβ‒DNA^dRP^ complex with *Sp*-dCTPαS

Crystals of hPolβ‒DNA^dRP^ were obtained as described previously [7]. The crystals were subsequently soaked with *Sp*-dCTPαS (2 mM) for 16 h in cryoprotectant buffer (0.05 M citrate, pH 5.5, 25% [w/v] PEG 6000, 15% [v/v] ethylene glycol, and 5 mM CaCl_2_). X-ray diffraction data were collected at 100 K on the LRL-CAT beamline at Advance Photon Source at Argonne National Laboratory (Lemont, IL) using a wavelength of 0.976 Å. Diffraction data were indexed and integrated with XDS [55] and merged and scaled using AIMLESS [56]. Low- and high-resolution cutoffs of 50 and 2.1 Å, respectively, were applied, yielding a final completeness of 90.88%. Initial phases were determined by molecular replacement using Phaser [57], with the hPolβ‒DNA^dRP^●dCTP structure (PDB: 7RBH) as the search model. Model building and refinement were performed using Coot [58] and REFMAC5 [59], respectively. The final atomic coordinates and the structure factors have been deposited in the Protein Data Bank under accession code 9Y62. Data collection and refinement statistics are summarized in Supplementary Table S2.

## Results

### Preparation of a stable and catalytically active crosslinked hPolβ‒DNA^dRP^ complex

The crosslinked hPolβ‒DNA^dRP^ complex was prepared according to our previously published procedures [7]. The procedures involved a partial BER reconstitution *in vitro* (Fig. 3A): a deoxyuridine-containing DNA^N^ substrate was successively treated with UDG to remove uracil, APE1 to cleave the resulting abasic site, and then hPolβ, followed by NaCNBH_3_ reduction to form the crosslinked hPolβ‒DNA^dRP^ complex (Materials and methods). hPolβ‒DNA^dRP^ was isolated from the reaction mixture by size-exclusion chromatography to remove free DNA^N^, unreacted NaCNBH_3_, uncrosslinked hPolβ, and other proteins. Notably, the 5′-terminus of the upstream primer within the purified hPolβ‒DNA^dRP^ complex can be [^32^P]-labeled for kinetic assays [7].

### The measured sulfur elemental effect suggests that the chemical step is not rate-limiting for correct nucleotide incorporation by crosslinked hPolβ

The sulfur elemental effect is a kinetic probe used to identify the rate-limiting chemical step in a nucleotide incorporation kinetic mechanism by substituting a non-bridging oxygen atom at the α-phosphate of an incoming dNTP for a less electronegative sulfur atom. This substitution alters the electron density of the P=O bond in the dNTP α-phosphate group, destabilizing the transition state during phosphodiester bond formation or cleavage and consequently decreasing the observed chemical reaction rate [20, 60]. For uncrosslinked DNA polymerases, reverse transcriptases, and RNA polymerases, comparison of the incorporation rates of dNTP and its *Sp*-dNTPαS analog has been widely used to probe the extent to which the chemical step (Step 4, Fig. 2) is rate-limiting during nucleotide incorporation [20, 21]. We applied this kinetic probe to examine if the chemical step limits dNTP incorporation by DNA-crosslinked hPolβ. To determine the sulfur elemental effect, a solution of [^32^P]-labeled hPolβ‒DNA^dRP^ (30 nM, Fig. 3A) was reacted with either dCTP (25 µM) or *Sp*-dCTPαS (25 µM) for various times before quenching with EDTA (Fig. 3B). Product-formation time courses from three independent experiments were fit individually to Equation 1, and the reported kinetic parameters represent the mean ± SD (*n* = 3). For dCTP incorporation, the observed rate constant (*k*_obs_) was found to be 0.77 ± 0.08 s^−1^ and the reaction amplitude (*A*) was 18.8 ± 0.6 nM. In contrast, for *Sp*-dCTPαS incorporation, the *k*_obs_ was 0.21 ± 0.01 s^−1^ and the *A* was 19.8 ± 0.2 nM (Fig. 3C). The sulfur elemental effect, calculated for each replicate as (*k*_obs,dCTP_/*k*_obs,dCTPαS_) and averaged, was 3.7 ± 0.4.

To evaluate whether crosslinking between hPolβ and the DNA substrate affected the sulfur elemental effect, we performed similar kinetic assays using equimolar amounts of uncrosslinked hPolβ and DNA^P^ (Supplementary Fig. S2C). A preincubated solution of free hPolβ (30 nM) and 5′-[^32^P]-labeled DNA^P^ (30 nM, Supplementary Fig. S4A) was reacted with either dCTP (25 µM) or *Sp*-dCTPαS (25 µM) for various times before quenching with EDTA. The product formation time courses (Supplementary Fig. S4B) were fit to Equation 1, yielding a *k*_obs_ of 0.11 ± 0.02 s^−1^ and an *A* of 16 ± 2 nM for dCTP incorporation, and 0.048 ± 0.003 s^−1^ and 3.5 ± 0.1 nM for *Sp*-dCTPαS incorporation. These *k*_obs_ values correspond to a sulfur elemental effect of 2.3 ± 0.4. The reduced reaction amplitude observed with uncrosslinked hPolβ can be attributed to a high DNA dissociation rate (0.93 s^−1^) measured previously [24] and the low *k*_obs_ (0.11 s^−1^), conditions not present in the DNA-crosslinked hPolβ. Interestingly, the sulfur elemental effect was similar for both uncrosslinked (2.3 ± 0.4) and crosslinked (3.7 ± 0.4) hPolβ, suggesting that crosslinking does not significantly alter the kinetic sensitivity of nucleotide incorporation to α-thio substitution. The measured small sulfur elemental effect of 3.7 ± 0.4 further suggests that correct dCTP incorporation by crosslinked hPolβ is unlikely rate-limited by phosphodiester bond formation [20, 60].

### The measured sulfur elemental effect suggests that the chemical step is likely rate-limiting for incorrect nucleotide incorporation by crosslinked hPolβ

The sulfur elemental effect associated with misincorporation was assessed using assays analogous to those employed for correct nucleotide incorporation (see above). In these experiments, a solution of [^32^P]-labeled hPolβ‒DNA^dRP^ (30 nM) was reacted with either incorrect dGTP (400 µM) or its α-thiol analog, *Sp*-dGTPαS (400 µM). We previously determined the dissociation equilibrium constant of incorrect dGTP with hPolβ‒DNA^dRP^ (*K*_d_ = 166 µM) [7]. To achieve sufficient dGTP misincorporation without significant Mg^2+^ chelation from the reaction buffer, a sub-saturating concentration of dGTP (400 µM) was utilized. Analysis of the dGTP misincorporation time course using Equation 1 yielded a *k*_obs_ of 5.2 ± 0.4 × 10^−4^ s^−1^ and an *A* of 23.8 ± 1.6 nM (mean ± SD, *n* = 3 independent experiments) (Fig. 3D). Due to the low product yield observed with *Sp*-dGTPαS misincorporation, the corresponding time course was analyzed by linear regression, giving a slope of 5.2 ± 0.5 × 10^−4^ nM/s. Assuming the reaction amplitude determined for dGTP misincorporation reflects the active enzyme concentration, the *k*_obs_ of *Sp*-dGTPαS misincorporation was calculated to be 2.2 ± 0.3 × 10^−5^ s^−1^. Consequently, the sulfur elemental effect for dGTP misincorporation was determined to be 24 ± 4. Comparable experiments performed using equimolar concentrations of uncrosslinked hPolβ and DNA^P^ (Supplementary Fig. S4A) with either dGTP or *Sp*-dGTPαS yielded negligible product under these conditions (data not shown), consistent with the high DNA dissociation rate (0.93 s^−1^) for the uncrosslinked enzyme [24] and the relatively slow misincorporation rates observed here. In conclusion, the large sulfur elemental effect (24 ± 4) suggests that dGTP misincorporation by crosslinked hPolβ is likely rate-limited by phosphodiester bond formation [20, 60].

### Ternary structure of hPolβ–DNA^dRP^●*Sp*-dCTPαS reveals minimal structural perturbation by the α-thio analog of dCTP

**Figure 4.**
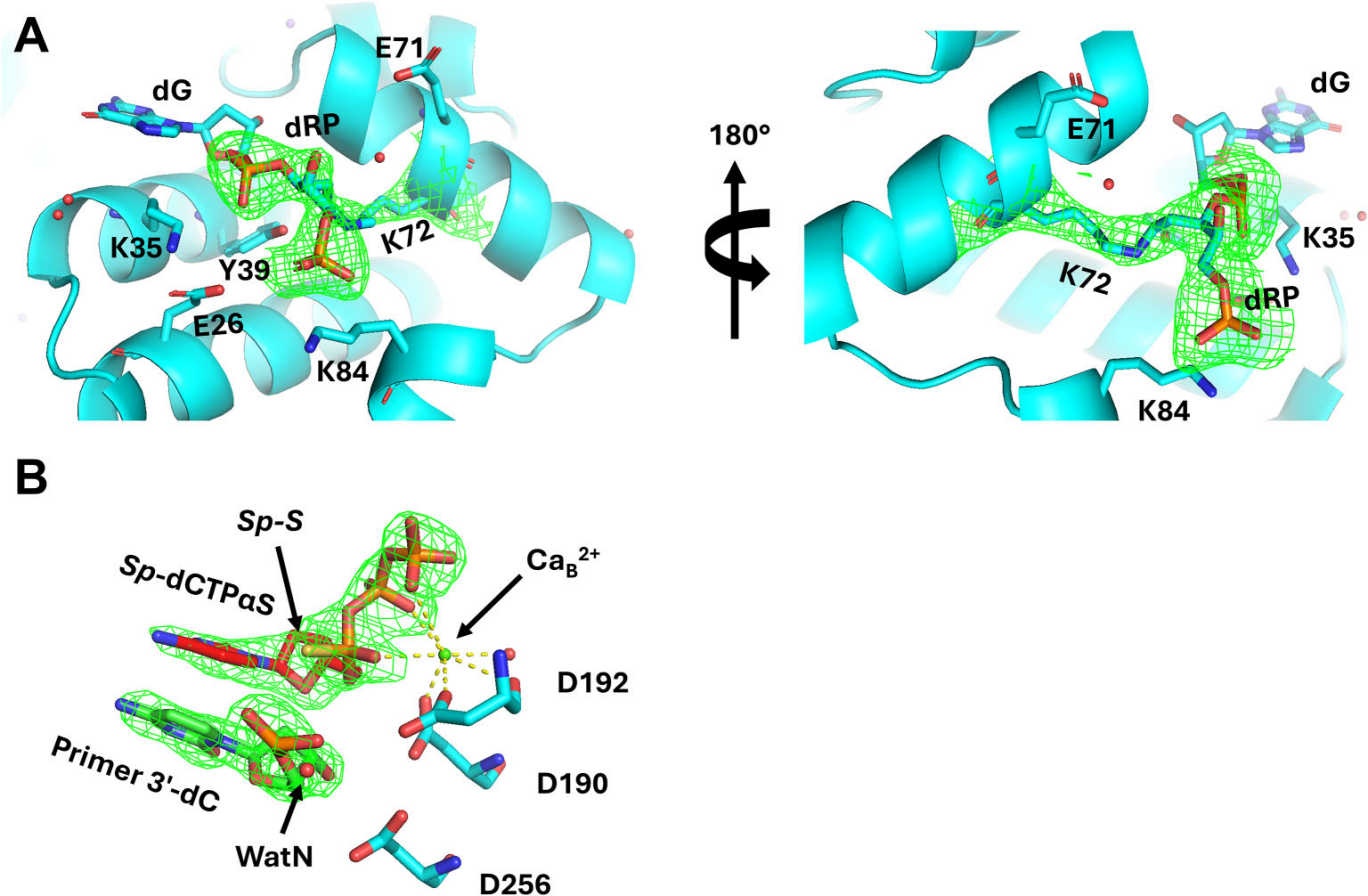
Ternary structure of hPolβ‒DNA^dRP^●*Sp*-dCTPαS (PDB: 9Y62). (**A**) Zoomed-in view of the dRP lyase active site showing the covalent crosslink between K72 and the dRP moiety. Two views are shown, including a 180° rotation of the same site. The 2*F_o_–F_c_* difference map (green, contoured at 1σ) is shown for the cross-linked and ring-opened dRP moiety. Water molecules are shown as small red spheres. (**B**) polymerase active site displaying the positioning of *Sp*-dCTPαS, whose base stacks with the primer 3′-dC, along with catalytic residues D190, D192, and D256, water molecules (red sphere) including WatN, and Ca^2+^ (green sphere) at the divalent metal ion binding B site. The 2*F_o_–F_c_* difference map (green, 1σ) is shown for *Sp*-dCTPαS and the primer 3′-dC. Coordination interactions are represented as dashed lines.

Crystals of the hPolβ‒DNA^dRP^ complex were obtained as described previously [7] and subsequently soaked with *Sp*-dCTPαS in the presence of the catalytically inactive divalent metal ion Ca^2+^. The structure of the resulting ternary complex (Fig. 4), hPolβ‒DNA^dRP^●*Sp*-dCTPαS, was determined by molecular replacement (Materials and methods) and refined to a resolution of 2.10 Å. The refined model is closely superimposable with our previously reported hPolβ‒DNA^dRP^●dCTP structure obtained in the presence of Ca^2+^ (PDB: 7RBH), with an overall RMSD value of 0.566 Å. As observed previously, the covalent crosslink between the K72 side chain and the ring-opened dRP moiety is clearly resolved within the dRP lyase active site (Fig. 4A), with continuous electron density supporting the linkage. *Sp*-dCTPαS is well defined in the polymerase active site (Fig. 4B), exhibiting clear 2*F_o_–F_c_* electron density for both the nucleotide and the coordinating metal ion. The *Sp* configuration positions the α-phosphorothioate sulfur away from the three catalytic aspartate residues and the divalent metal ion at the B site, consistent with the reported structure of uncrosslinked hPolβ●DNA^P^●*Sp*-dCTPαS (PDB: 5U9H) [61]. Only a single divalent metal ion is observed in the polymerase active site, with Ca²⁺ occupying the B site. This arrangement is consistent with an early ternary binding state similar to (hPolβ‒DNA^dRP^●dCTP)_1_ structure (PDB: 7RBG), in which a Na^+^ ion occupies the A site while a Ca^2+^ ion is present at the B site. Together, these observations indicate that the hPolβ‒DNA^dRP^●*Sp*-dCTPαS structure represents a pre-chemistry ternary state with nucleotide positioning closely matching that of native complexes, supporting its use for mechanistic interrogation of nucleotide incorporation.

### Viscosity independence rules out large-scale domain motions as rate limiting

**Figure 5.**
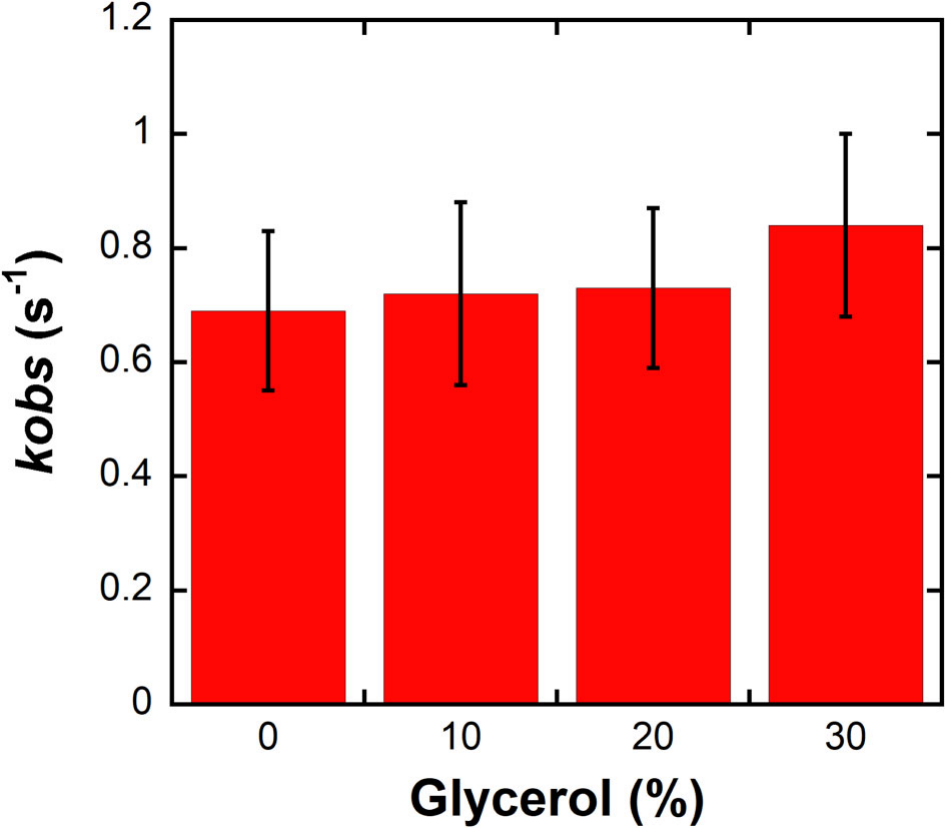
Effect of solution viscosity on the nucleotide-saturated rate constant of correct dCTP incorporation by DNA crosslinked hPolβ. A solution of [^32^P]-labeled hPolβ‒DNA^dRP^ (30 nM) was rapidly mixed with dCTP (25 µM) in reaction buffer containing increasing concentrations of glycerol (0%–30% v/v) and incubated for varying times prior to quenching with EDTA. Product-formation time courses at each glycerol concentration were analyzed by nonlinear regression using Equation 1 to determine *k*_obs_ values, which are plotted with their associated standard errors.

Variation of solvent viscosity has been used to assess whether large-scale protein motions kinetically limit enzymatic reactions [30]. To assess the effect of solvent viscosity on nucleotide incorporation, a solution of [³²P]-labeled hPolβ–DNA^dRP^ (30 nM) was reacted with dCTP (25 µM) in the presence of increasing concentrations of glycerol from 0% to 30% (v/v) for various times before quenching with EDTA. Product formation was quantified from these time courses and fit to Equation 1 (Materials and methods). The observed reaction rate constant remained unchanged within the experimental error across the full range of glycerol concentrations tested (Fig. 5). These results indicate that large-scale protein motions are unlikely to be rate-limiting for hPolβ-catalyzed dCTP incorporation [30].

### Pulse-chase and pulse-quench amplitude differences reveal a kinetically significant ternary intermediate preceding the chemical step

**Figure 6.**
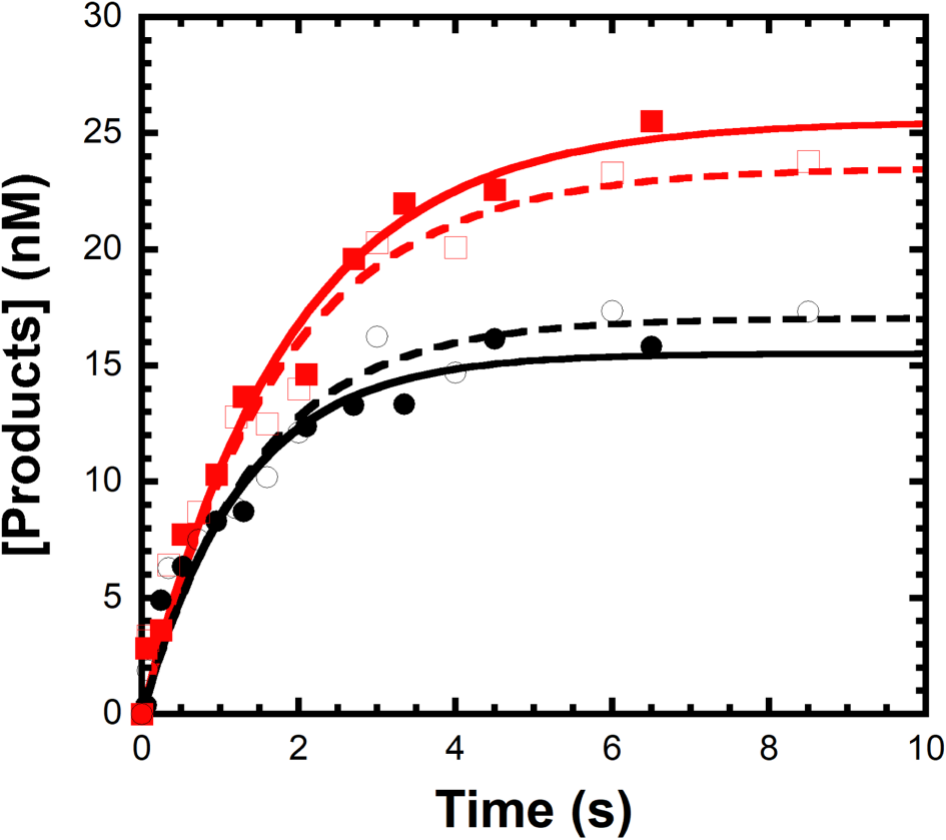
Pulse-quench pulse-chase assays with DNA-crosslinked hPolβ. A solution of unlabeled hPolβ‒DNA^dRP^ was rapidly mixed with [α-^32^P]dCTP (1.5 µM) for different times. For the two independent pulse-quench assays, the reactions were rapidly quenched with 1 M HCl (● and ○). For the two independent pulse-chase assays, the reactions were chased with 1.5 mM cold dCTP (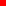 and 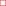) for 20 s prior to quenching with 1 M HCl. Product formation time courses were fit individually to Equation 1 (Materials and methods), and reported values represent the mean ± SD from two independent experiments (*n* = 2). The pulse–quench reaction yielded an amplitude of 16.3 ± 1.1 nM with a *k*_obs_ of 0.74 ± 0.07 s^−1^, whereas the pulse-chase reaction produced a higher amplitude of 24.5 ± 1.4 nM and a slightly lower *k*_obs_ 0.55 ± 0.02 s^−1^.

For uncrosslinked polymerases, the difference in reaction amplitudes between pulse-quench and pulse-chase assays has been commonly used to identify the rate-limiting protein conformational change that precedes the chemical step during nucleotide incorporation [20, 21]. To probe if a slow protein conformational change step limits gap-filling DNA synthesis by DNA-crosslinked hPolβ, we performed pulse-quench and pulse-chase experiments. In these experiments, a solution containing unlabeled hPolβ‒DNA^dRP^ (30 nM) was first rapidly mixed with [α-^32^P]dCTP (1.5 µM) for various times. In the pulse-quench assay, addition of 1 M HCl instantly quenched the reaction. In the pulse-chase assay, 1000-fold excess of unlabeled dCTP was added to the reaction mixture for 20 s followed by quenching with 1 M HCl. Strong acid quenched reactions were partitioned with chloroform to denature the enzyme, and the aqueous layer was rapidly neutralized with 1 M NaOH prior to analysis by Urea‒PAGE. In contrast to the pulse-quench assay, where all enzyme-bound species were instantly quenched, the 20 s chase (∼17 half-lives) allowed partitioning of the hPolβ‒DNA^dRP^ and dCTP ternary complexes in both the forward and reverse directions. During this chase, stable, productive complexes were chased by the 1000-fold excess unlabeled dCTP, which promoted the partitioning of radiolabeled ternary complexes in the forward direction, resulting in an increase in observed DNA product formation. Consequently, a difference in the reaction amplitudes between the pulse-quench and pulse-chase assays provides direct evidence for an enzyme-bound ternary complex preceding the chemical step as previously demonstrated with uncrosslinked polymerases [20, 52, 53, 62]. Product-formation time courses from two independent experiments were fit individually to Equation 1, and kinetic parameters represent the mean ± SD (*n* = 2). The pulse-quench assay yielded an *A* of 16.3 ± 1.1 nM and a *k*_obs_ of 0.74 ± 0.07 s^−1^, whereas the pulse-chase assay yielded an *A* of 24.5 ± 1.4 nM and a *k*_obs_ of 0.55 ± 0.02 s^−1^ (Fig. 6). The resulting amplitude ratio (*A*_pulse-chase_/*A*_pulse-quench_) was calculated to be 1.5 ± 0.1, providing compelling evidence for an enzyme-bound ternary complex with crosslinked hPolβ that was chased forward to form additional product.

### Eyring analysis reveals a high activation free energy barrier for nucleotide incorporation

**Figure 7.**
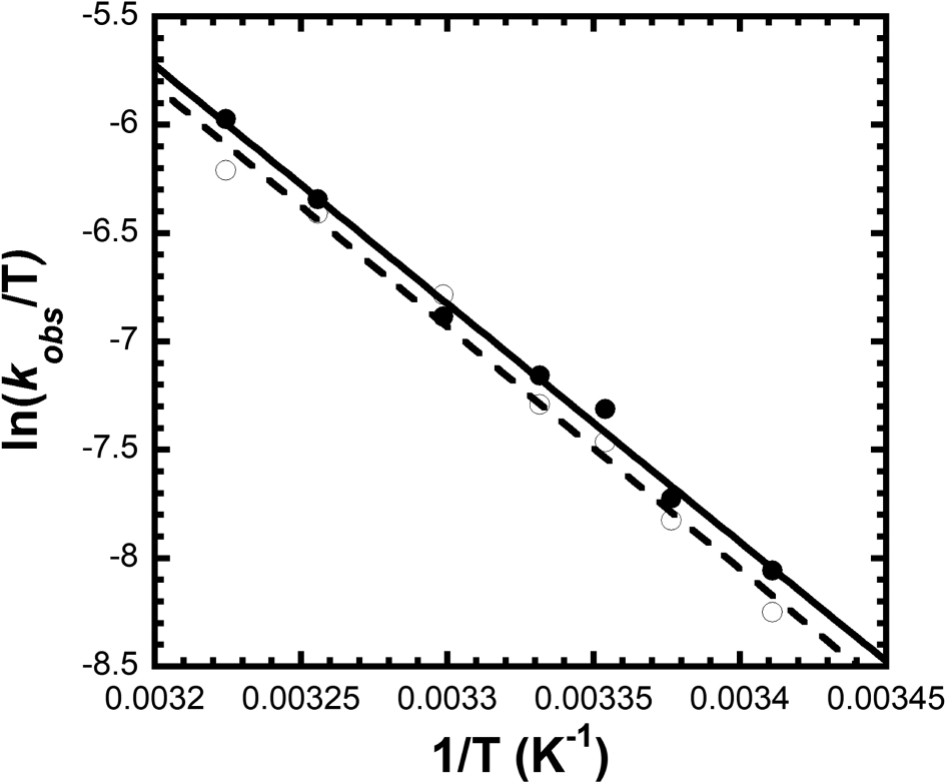
Eyring plot showing the temperature dependence of the nucleotide-saturated incorporation rate constant for correct dCTP incorporation by crosslinked hPolβ. A solution of radiolabeled hPolβ‒DNA^dRP^ (30 nM) was rapidly mixed with dCTP (25 µM) for different times to generate product-formation time courses at temperatures ranging from 20 to 37°C. The *k*_obs_ obtained from fitting these time courses were analyzed using the linearized Eyring equation. Data from two independent replicates (● and ○) are shown, with solid and dotted lines indicating the corresponding linear fits.

To determine the activation parameters for correct nucleotide incorporation catalyzed by crosslinked hPolβ, single-turnover kinetic assays were performed over a range of temperatures under nucleotide-saturating conditions. A solution containing [^32^P]-labeled hPolβ‒DNA^dRP^ (30 nM) was rapidly mixed with dCTP (25 µM) for various times and quenched with EDTA. The equilibrium dissociation constant (*K*_d_) for dCTP binding to hPolβ‒DNA^dRP^ was previously determined to be 0.38 µM [7], confirming that 25 µM dCTP provides saturating conditions, such that *k*_obs_ approximates the maximal nucleotide incorporation rate *k*_p_. Product formation was monitored over a temperature range of 20 to 37°C. At each temperature, the corresponding time courses were independently fit to Equation 1 (Materials and methods) to obtain *k*_obs_ values. The temperature dependence of these *k*_obs_ values was then analyzed using the linearized Eyring equation (Equation 2, Materials and methods), as shown in Fig. 7. From two independent experiments, the activation parameters for dCTP incorporation were determined to be $\vartriangle {{H}^\ddagger }$ = 21.5 ± 0.2 kcal/mol and ${\mathrm{\Delta }}{{S}^\ddagger }\ $= 11.5 ± 0.5 cal/mol⋅K (mean ± SD, *n* = 2). These values were used to determine ${\mathrm{\Delta }}{{G}^\ddagger }$ = 18.0 ± 0.3 kcal/mol at 37°C, where uncertainty in ${\mathrm{\Delta }}{{G}^\ddagger }$ was calculated by error propagation from $\vartriangle {{H}^\ddagger }$ and ${\mathrm{\Delta }}{{S}^\ddagger }$. The relatively large$\ {\mathrm{\Delta }}{{G}^\ddagger }$ indicates that crosslinked hPolβ must overcome a substantial energy barrier to catalyze dCTP incorporation.

## Discussion

The rate-limiting step for correct nucleotide incorporation by uncrosslinked Polβ has remained debated for decades, with evidence supporting contributions from protein conformational transitions and/or the phosphodiester bond formation step [15, 19–21, 24–26]. The covalently crosslinked Polβ–DNA intermediate resulting from Schiff base reaction to an abasic site provides an opportunity to examine this question under conditions that more closely reflect the physiological BER pathway [7]. By integrating pre-steady-state kinetic analysis with previously reported structural characterization of the crosslinked Polβ–DNA complex [7] and a newly solved structure (Fig. 4), we sought to define the kinetic mechanism of nucleotide incorporation and determine whether the rate-limiting step arises from phosphodiester bond formation and/or from pre-chemical active-site reorganization within a tightly bound ternary complex.

### Biological relevance of DNA crosslinking of Polβ and several other DNA polymerases

Previous mechanistic studies have relied on chemically stable but biologically irrelevant DNA substrates (Supplementary Fig. S2A–D) to circumvent the innate instability of the dRP moiety [15, 44, 63, 64]. These analogs, however, preclude the formation of a covalent crosslink between the dRP lyase domain of Polβ and the DNA substrate as required by BER (Supplementary Fig. S1). With a physiologically relevant substrate DNA^dRP^ (Supplementary Fig. S2E) containing an *in situ* generated abasic site by the first two BER enzymes (Supplementary Fig. S1), we have recently redefined the BER pathway, demonstrating that hPolβ’s dRP lyase and gap-filling DNA polymerase activities proceed through a three-step process: Schiff base formation, gap-filling DNA synthesis, and finally, β-elimination of the dRP moiety (Supplementary Fig. S1). This revised pathway indicates that the polymerase domain of hPolβ incorporates a nucleotide when the enzyme is covalently linked to a single-nucleotide gapped DNA substrate through Schiff base formation between Lys72 within the dRP lyase active site and the opened-ring aldehyde of the dRP moiety at an *in situ* generated abasic site (Fig. 1B and Supplementary Fig. S3) [24].

Relative to uncrosslinked hPolβ, the DNA-crosslinked state eliminates rapid DNA dissociation, binds correct dCTP with a ∼70-fold lower *K*_d_, incorporates dCTP with a 4-fold lower *k*_p_, and increases dCTP incorporation efficiency (*k*_p_/*K*_d_) by ∼20-fold [24]. This kinetic profile suggests that DNA crosslinking stabilizes the polymerase–DNA complex, reducing conformational sampling while maintaining catalytic efficiency. Interestingly, DNA polymerases λ (Polλ) [65], γ [66], ι [67, 68], and θ [69] as well as Rev1 [70] also possess both dRP lyase and polymerase activities *in vitro* raising the possibility that they may employ similar strategies to enhance DNA polymerization *in vivo*. For example, human Polλ, an X-family homolog of hPolβ, incorporates a correct nucleotide at a *k_p_* of 3 to 6 s^−1^ but exhibits relatively rapid DNA dissociation (0.8 s^−1^) in the absence of cofactors [71]. In the context of non-homologous end-joining during double-strand break repair, interaction of Polλ with Ku proteins enhances both DNA binding and the rate of nucleotide incorporation, thereby improving its effectiveness *in vivo* [72]. In contrast, Polλ functions as a backup polymerase in BER, where strong DNA end-binding cofactors are absent [71]; thus, alternative mechanisms to stabilize the polymerase–DNA complex may be required. DNA crosslinking could represent one such mechanism; however, its contribution to DNA binding or complex stability in these systems remains to be established. More broadly, covalent tethering may represent a general kinetic strategy among repair polymerases to maintain processivity in systems that otherwise exhibit high DNA turnover.

### Proposed kinetic mechanism of DNA synthesis catalyzed by a DNA-crosslinked polymerase

**Figure 8.**
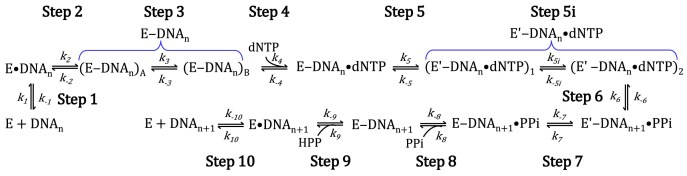
Proposed minimal kinetic mechanism for DNA-crosslinked hPolβ. E●DNA_n_ and E●DNA_n+1_ refer to the binary complexes of uncrosslinked hPolβ with DNA_n_ and DNA_n+1_, respectively. E‒DNA_n_ refers to the crosslinked Polβ‒DNA^dRP^ complex after Schiff base formation but before β-elimination. E and E′ represent open and closed conformations of hPolβ, respectively. PPi and HPP refer to pyrophosphate and *cis-*4-hydroxy-2-pentenal-5-phosphate, respectively.

While a general minimal kinetic mechanism for DNA synthesis has been established for many uncrosslinked polymerases [20, 52, 53, 62, 73–78], including uncrosslinked hPolβ by us recently (Fig. 2) [24], a detailed kinetic mechanism for nucleotide incorporation catalyzed by DNA-crosslinked polymerases has never been established. This mechanism requires revision for DNA-crosslinked hPolβ, as the complete collapse of E●DNA_n_●dNTP and E′●DNA_n_●dNTP (*k_8_* and *k_9_*, Fig. 2) does not occur while the enzyme and substrate are crosslinked. Based on our prior kinetic and structural studies of DNA-crosslinked hPolβ [7], we propose a modified kinetic mechanism for a DNA-crosslinked polymerase (Fig. 8).

**Formation and equilibration of DNA-crosslinked hPolβ binary complexes**

**Figure 9.**
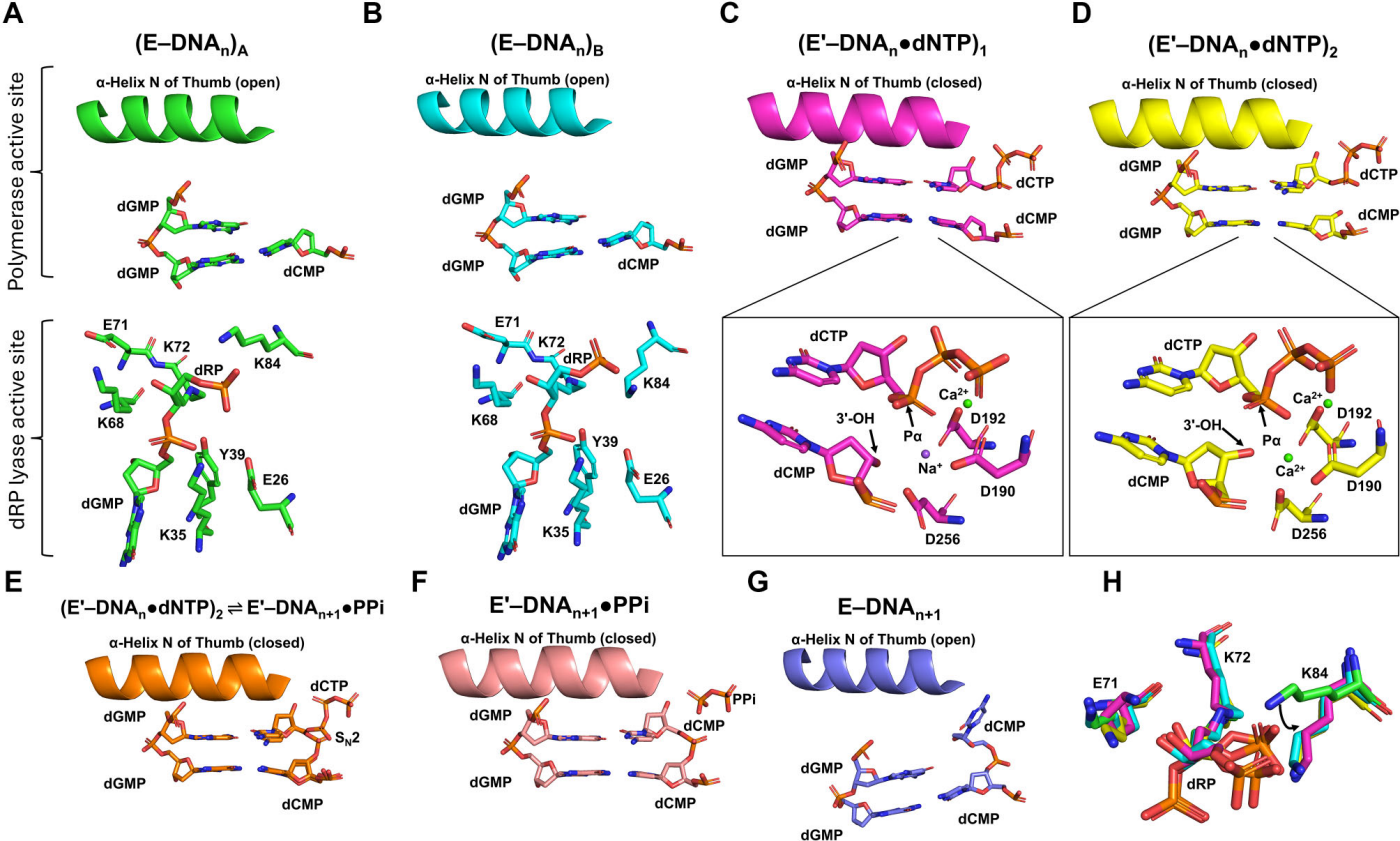
Structural evidence [7] for the existence of the species included in the kinetic mechanism for DNA-crosslinked hPolβ (Fig. 8). (**A**) The binary crystal structure (hPolβ‒DNA^dRP^)_A_ (PDB: 7RBE) representing (E‒DNA_n_)_A_. (**B**) The binary crystal structure (hPolβ‒DNA^dRP^)_B_ (PDB: 7RBF) indicating (E‒DNA_n_)_B_. (**C**) The ternary crystal structure (PDB: 7RBG) representing (E'‒DNA_n_●dNTP)_1_ with the upstream primer 3′-OH pointing away from the dCTP Pα and a distance of 5.4 Å between them. (**D**) The ternary crystal structure (PDB: 7RBH) denoting (E'‒DNA_n_●dNTP)_2_ with the upstream primer 3′-OH pointing toward the dCTP Pα and a distance of 3.7 Å between them. (**E**) Reaction-state structure (PDB: 7RBJ) of 50% dCTP incorporation demonstrates (E'‒DNA_n_●dNTP)_2_ ⇌ E'‒DNA_n+1_•PPi (Step 6). The Pα steric configuration inversion and S_N_2 reaction are shown. (**F**) Post-catalytic structure (PDB: 7RBL) indicating E'‒DNA_n+1_●PPi. (**G**) Post-catalytic structure (PDB: 7RBN) representing E‒DNA_n+1_. (**H**) Superposition of zoomed dRP active sites in the binary and ternary structures (PDB: 7RBE, 7RBF, 7RBG, and 7RBH). The K84 position in (E‒DNA_n_)_A_ (green) differs substantially from the other three structures, with a displacement (arrow) of ∼4.2 Å. In panels (A) to (G), the open or closed polymerase domain is indicated by open or closed α-Helix N, respectively.

Following processing of single-base damaged DNA by the first two BER enzymes (Supplementary Fig. S1), the *in situ* generated DNA intermediate DNA^dRP^ (Supplementary Fig. S2E) initially binds noncovalently to the polymerase domain of hPolβ (Step 1, Fig. 8) and then forms a Schiff base with the dRP lyase domain (Fig. 1B). Two binary structures of DNA-crosslinked hPolβ, (hPolβ‒DNA^dRP^)_A_ (Fig. 9A) and (hPolβ‒DNA^dRP^)_B_ (Fig. 9B) [7] designated (E‒DNA_n_)_A_ and (E‒DNA_n_)_B_ (collectively referred to as E‒DNA_n_ in Fig. 8), contain only minor differences, including a change in the rotameric configuration of Lys84 and a slight shift in the position of the crosslinked dRP moiety within the dRP lyase active site (Fig. 9H). Both binary structures exhibit an open polymerase domain, as indicated by the positioning of α-Helix N [7], are formed via Schiff base formation (Step 2, Fig. 8), and are likely in equilibrium (Step 3, Fig. 8). Comparing the two binary structures, (hPolβ‒DNA^dRP^)_B_ is presumed to be formed later, as its Lys84 residue aligns well with the same residue in the E′‒DNA_n_●dNTP ternary structures (Fig. 9H) (see below).

**A pre-catalytic ternary intermediate undergoes structural alignment prior to the chemical step**

Following correct dCTP binding (Step 4, Fig. 8), we have previously obtained two pre-catalytic ternary structures, (hPolβ‒DNA^dRP^●dNTP)_1_ (Fig. 9C) and (hPolβ‒DNA^dRP^●dNTP)_2_ (Fig. 9D) [7]. (hPolβ‒DNA^dRP^●dNTP)_1_ is considered a precursor to (hPolβ‒DNA^dRP^●dNTP)_2_, as its upstream primer 3′-OH points away from the dCTP α-phosphate (Pα) (Fig. 9C) and the distance between them is longer by 1.7 Å [7], preventing nucleophilic attack. In (hPolβ‒DNA^dRP^●dNTP)_2_, this misalignment is corrected, suggesting a structural change that prepares the ternary complex for catalysis (Fig. 9D). Consistently, (hPolβ‒DNA^dRP^●dNTP)_2_ and all reaction-state structures, e.g. structure in Figs 1C and 9E, solved through our time-resolved X-ray crystallography, position DNA^dRP^ and dCTP in a catalytically productive configuration for catalysis [7]. Because both (hPolβ‒DNA^dRP^●dNTP)_1_ and (hPolβ‒DNA^dRP^●dNTP)_2_, like the reaction-state structures, exhibit polymerase domain closure as exemplified by the closure of α-Helix N [19] (Fig. 9), they are kinetically designated as (E′‒DNA_n_●dNTP)_1_ and (E′‒DNA_n_●dNTP)_2_, respectively, and collectively referred to as E′‒DNA_n_●dNTP (Fig. 8). As part of Step 5, we designate the structural transition from (E′‒DNA_n_●dNTP)_1_ to (E′‒DNA_n_●dNTP)_2_ as Step 5i (Fig. 8). Importantly, these structures validate the existence of two distinct closed states – one poised for alignment and one competent for catalysis, directly linking structural rearrangements to the kinetic step we identify as rate-limiting (see below).

**Phosphodiester bond formation proceeds via an S_N_2 mechanism in a closed active site**

During the chemical step (Step 6, Fig. 8), the upstream primer 3′-OH attacks the dCTP Pα, forming a new phosphodiester bond and simultaneously breaking the dCTP α-β phosphoanhydride bond (Figs 1C and 9E) [7]. To facilitate the nucleophilic attack, a water molecule (WatN), part of a hydrogen-bonded network, accepts a proton from the upstream primer 3′-OH and transfers it to the solvent (Fig. 1C and D) [7, 79]. This phenomenon has also been observed with DNA polymerase η [80]. These features, along with the steric inversion of the Pα stereocenter, are consistent with an S_N_2 reaction (Figs 1C and 9E). Additionally, our time-resolved reaction-state structures are almost superimposable and contain a closed protein conformation [7] (Fig. 9E).

**Domain reopening precedes pyrophosphate release in post-catalytic steps**

Following complete phosphodiester bond formation (Step 6), hPolβ remains in the closed conformation with PPi bound as shown in the post-catalytic structure of E′‒DNA_n+1_ (Fig. 9F). Interestingly, we have also solved the post-catalytic structure of E‒DNA_n+1_ (Fig. 9G), showing that DNA-crosslinked hPolβ adopts an open conformation after PPi dissociates [7], reflecting the net results of Steps 7 and 8 (Fig. 8). Although we were unable to capture the post-catalytic structure of E‒DNA_n+1_●PPi, our published structural studies with uncrosslinked hPolβ reveal that the reverse protein conformational change occurs prior to PPi dissociation (Step 5, Fig. 2) [81]. This order is presumed to apply to DNA-crosslinked hPolβ as well, so that Step 7 precedes Step 8 in our proposed kinetic mechanism (Fig. 8). Notably, our prior kinetic studies [7] have shown that β-elimination by the dRP lyase activity occurs after gap-filling DNA synthesis, resulting in the release of *cis-*4-hydroxy-2-pentenal-5-phosphate (HPP) and formation of a non-covalent binary complex of hPolβ and the nicked DNA product (E●DNA_n+1_) (Step 9, Fig. 8). Finally, uncrosslinked hPolβ dissociates from the nicked product (Step 10, Fig. 8). Rather than dissociating and risking degradation by cellular nucleases, the nicked DNA_n+1_ product is likely transferred directly from hPolβ to a human DNA ligase for binding and ligation, completing the last step of BER (Supplementary Fig. S1).

**Kinetic parameters for the proposed mechanism of DNA-crosslinked hPolβ**

For the proposed kinetic mechanism in Fig. 8, our prior work [7] has established the following parameters at 25°C (Supplementary Table S3): a Schiff base formation rate constant (*k*_2_) of 4.5 s^−1^, a *K*_d_ (*k*_-4_/*k*_4_) of 0.38 µM for dCTP binding, a correct dCTP incorporation rate constant (*k*_5_ = *k*_p_) of 0.72 s^−1^, and a β-elimination rate constant of 0.31 s^−1^. We further fit the time-dependent disappearance of hPolβ–[^32^P]–HPP [7] and estimated *k_9_* (Fig. 8) to be ∼0.14 s^−1^ (data not shown). The microscopic rate constants *k*_2_, *k*_5_, and *k*_9_ are expected to be higher at 37°C. Using DNA^p^ as a substrate (Supplementary Fig. S2C), we have previously determined *k*_7_ (0.93 s^−1^) and estimated *k*_-1_ (∼0.93 s^−1^) for uncrosslinked hPolβ at 37°C (Fig. 2) [24]. Given the similarity between DNA^p^ (Supplementary Fig. S2C) and DNA^dRP^ (Supplementary Fig. S2E), it is reasonable to assume *k*_-1_ and *k*_10_ in Fig. 8 are also close to 0.93 s^−1^ (Supplementary Table S3). These measured kinetic parameters, together with the estimated *k*_6_/*k*_-6_ ratio for Step 6 (see below), are summarized in Supplementary Table S3.

### Correct nucleotide incorporation by DNA-crosslinked hPolβ is likely limited by a pre-chemical step

To identify the rate-limiting step for correct nucleotide incorporation catalyzed by DNA-crosslinked hPolβ (Fig. 8), we obtained four lines of evidence (Supplementary Table S3), as described below:

**The modest sulfur elemental effect indicates that the chemical step is not rate-limiting for correct nucleotide incorporation**

The sulfur elemental effect, a widely employed kinetic probe for identifying the rate-limiting step in the kinetic mechanisms of DNA polymerases, reverse transcriptases, and RNA polymerases (Supplementary Table S1), involves substituting a non-bridging oxygen atom in the incoming dNTP’s α-phosphate with a less electronegative sulfur atom. This substitution destabilizes the transition state of phosphodiester bond formation (Supplementary Fig. S5B), reducing both forward (*k*_4_) and reverse (*k*_-4_) rate constants (Fig. 2). However, accurate interpretation of this sulfur elemental effect requires knowledge of the nature of the transition state (associative, dissociative, or intermediate). Recent time-resolved crystallographic studies of uncrosslinked hPolβ [82, 83], hPolη [84, 85], and hPolμ [86] have challenged the two metal-ion mechanism dogma [87], revealing a three metal-ion mechanism for nucleotide incorporation. These structural studies have also demonstrated concerted phosphodiester bond formation between the primer 3′-OH and dNTP’s Pα and the breakage of the dNTP α-β phosphoanhydride bond, coupled with the Pα stereocenter inversion, in line with an S_N_2 reaction mechanism (Supplementary Fig. S5A). Consistent with this S_N_2 reaction, we have proposed this transition state model for Step 4 (Fig. 2 and Supplementary Fig. S5B) [24].

For the S_N_2 reaction, analogous to phosphate triester hydrolysis [60], we have recently revised the benchmark for the sulfur elemental effect in the rate-limiting Step 4 (Fig. 2) to 10–160 for uncrosslinked polymerases [24]. This contrasts with the previously proposed range of 4 to 11 [88], a “gold standard” used in the field for over three decades. This revised benchmark suggests Step 4 (Fig. 2) is not rate-limiting for correct nucleotide incorporation by uncrosslinked polymerases (Supplementary Table S1). For incorrect nucleotide incorporation, the reported sulfur elemental effects for different polymerases (Supplementary Table S1) exhibit substantial variation but are generally large, suggesting rate-limiting Step 4 (Fig. 2). The inaccuracy of the reported sulfur elemental effects for incorrect nucleotides may result from slow misincorporation, fast DNA or RNA dissociation, and/or low nucleotide binding affinity. Notably, the sulfur elemental effects for correct and incorrect nucleotide incorporation by uncrosslinked hPolβ have been determined to be 3.94 and 64.6, respectively [24].

Intriguingly, like uncrosslinked hPolβ (Supplementary Fig. S5A) [82, 83], our time-resolved crystallographic structures of DNA-crosslinked hPolβ [7], such as the reaction-state structures (Figs 1C and 9E), also show the S_N_2 reaction mechanism for correct dCTP incorporation with hPolβ‒DNA^dRP^. We proposed a similar transition state model (Fig. 1D), suggesting the crosslinking between the downstream primer and the dRP lyase domain of hPolβ does not affect the chemical step. Thus, Step 6 (Fig. 8) likely follows the diagnostic sulfur elemental effect benchmark established for uncrosslinked polymerases [24]. Experimentally, DNA-crosslinked hPolβ yielded sulfur elemental effects of 3.7 ± 0.4 for dCTP incorporation (Fig. 3C) and 24 ± 4 for dGTP misincorporation (Fig. 3D). The comparable sulfur elemental effects of crosslinked and uncrosslinked hPolβ for dCTP incorporation confirm crosslinking does not affect phosphodiester bond formation. Therefore, comparison to the revised benchmark, Step 6 (Fig. 8) is likely not rate-limiting for correct (Supplementary Table S3), but is for incorrect dNTP incorporation by DNA-crosslinked hPolβ.

To confirm the nucleotide analog did not disrupt the active site of the crosslinked complex, crystals of the complex were soaked with the catalytically inactive divalent metal, Ca^2+^, and the nucleotide analog as described previously [6]. We then determined the crystal structure of the resulting ternary complex hPolβ‒DNA^dRP^●*Sp*-dCTPαS (Fig. 4). The position of the active site aspartate side chains in this structure is consistent with the pre-catalytic ternary structure previously observed with the natural nucleotide (Fig. 9A). Notably, the *Sp* orientation of the sulfur atom positions it away from catalytic residues and divalent metal ion at the B site, consistent with previous findings for uncrosslinked Polβ [61]. The active site contains a single divalent metal ion, which suggests an early intermediate state, resembling intermediate (E′‒DNA_n_●dNTP)_1_ described above. In this comparable structure, a sodium ion is observed where the divalent metal ion at the A site ultimately binds, aiding to position and abstract the proton from the 3′-OH of the templating nucleotide for catalysis. A previous criticism of sulfur elemental effect analysis is that the larger sulfur atom could introduce steric disruption within the active site, which has been proposed to account for the absence of a third divalent metal ion in structures of uncrosslinked Polβ and Polη [61, 85]. Although this concern is valid, the third divalent metal ion is often difficult to observe due to its transient nature and has remained elusive for decades. Therefore, its absence in time-resolved crystallographic experiments using the α-thio nucleotide derivative does not constitute conclusive evidence of steric interference.

**Lack of viscosity dependence indicates that large-scale conformational changes are not rate limiting**

Because the modest sulfur elemental effect suggests that the chemical step is unlikely to be the rate-limiting step, we next tested whether diffusion-dependent conformational motions limit the observed rate. Solution viscosity has been used to assess whether large-scale protein domain movements contribute to the observed rate [30]. In stopped-flow fluorescence studies of uncrosslinked Polβ, increasing the concentration of viscogens such as glycerol and sucrose selectively slowed a fast fluorescence transition that was interpreted as a protein conformational change occurring prior to catalytic metal-ion binding, whereas later fluorescence phases associated with chemistry were less sensitive to viscosity [30]. Because DNA crosslinking could potentially restrict Polβ domain mobility, we examined the effect of viscosity on nucleotide incorporation by the hPolβ–DNA^dRP^ complex. However, increasing the glycerol concentration from 0% to 30% (v/v) produced no measurable change in the observed incorporation rate under single turnover conditions. The absence of viscosity dependence indicates that large-scale protein motions, such as finger subdomain closing (Step 4 in Fig. 8), remain rapid relative to the observed rate and therefore are not rate limiting (Supplementary Table S3). Together with the modest elemental effect, these results constrain the rate-limiting step to local active-site rearrangements that are insensitive to bulk solvent viscosity yet precede phosphodiester bond formation.

**Pulse-chase and pulse-quench analyses identify a pre-chemical rate-limiting conformational change**

To directly evaluate whether a pre-chemical conformational transition limits catalysis, we employed pulse-chase and pulse-quench assays (Fig. 6), reliable methods for identifying the rate-limiting step in a polymerase kinetic mechanism (Supplementary Table S1) [20, 21]. A lower product yield in pulse-quench versus pulse-chase assays provides direct evidence for the existence of a tightly bound ternary complex prior to the chemical step and indicates a rate-limiting protein conformational change step preceding the chemical step [20, 21]. In our experiments, pulse-quench reactions were quenched immediately with strong acid, while pulse-chase reactions included a 20 s chase with 1000-fold excess unlabeled dCTP before quenching (Fig. 6). The significant difference (8.2 nM or 33%) in reaction amplitude between pulse-quench (16.3 ± 1.1 nM) and pulse-chase (24.5 ± 1.4 nM) assays strongly suggests a tightly bound intermediate complex forms before Step 6, and that Step 5 is rate-limiting (Fig. 8 and Supplementary Table S3).

Mechanistically, the intermediate complex could be E‒DNA_n_, E‒DNA_n_●dNTP, or E′‒DNA_n_●dNTP (Fig. 8). However, E‒DNA_n_ is ruled out because, during the chase, the binary complex hPolβ‒DNA^dRP^ would be 1000 times more likely to react with unlabeled dCTP, making it effectively undetectable and precluding any difference in reaction amplitudes. E‒DNA_n_●dNTP is also unlikely due to the kinetically slow dissociation rate of [α-^32^P]dCTP from this loosely bound ternary complex E‒DNA_n_●[α-^32^P]dCTP based on the following rationale: given the 33% reaction amplitude difference, the intermediate complex must partition between product formation (67%, 0.55 s^−1^, Fig. 6) and [α-^32^P]dCTP release (0.27 s^−1^, calculated), much slower than typical dNTP dissociation rates, e.g. 118 s^−1^ with uncrosslinked hPolβ [24]. Thus, this intermediate complex is most likely E′‒DNA_n_●dNTP (24.5 – 16.3 = 8.2 nM). Similar reasoning has previously demonstrated the existence of E′●DNA_n_●dNTP and a rate-limiting protein conformational change with uncrosslinked polymerases (Supplementary Table S1). This conclusion is further supported by our crystallographic studies of DNA-crosslinked hPolβ with correct dCTP, which have yielded only two closed pre-catalytic structures, (hPolβ‒DNA^dRP^●dNTP)_1_ and (hPolβ‒DNA^dRP^●dNTP)_2_, and have not revealed any open pre-catalytic ternary structures [7].

Moreover, the significant difference in reaction amplitudes between the pulse-quench and pulse-chase assays indicates a kinetic roadblock where Steps 5 and 7 (Fig. 8) are slower than Step 6 (Fig. 8) [21]. The internal equilibrium constant for Step 6 (*k*_6_/*k*_-6_ = 16.3 nM/8.2 nM ≅ 2, Supplementary Table S3) can be calculated from E′‒DNA_n+1_●PPi abundance (16.3 nM) and the reaction amplitude difference (8.2 nM). For comparison, the internal equilibrium constant for Step 4 (Fig. 2) with uncrosslinked hPolβ is 0.89 [24], meaning DNA crosslinking enhances the forward flux of the tight ternary complex toward product formation (Step 6, Fig. 8) by >2-fold.

**Eyring analysis indicates that the chemical step is not rate-limiting for correct nucleotide incorporation**

Consistent with the pulse–chase analysis indicating a kinetically significant pre-chemical step, Eyring analysis provides complementary insight into the energetic landscape governing nucleotide incorporation by hPolβ. For uncatalyzed phosphodiester bond formation in solution, the activation free energy for its rate-limiting chemical step is calculated to be 21.1 kcal/mol [89]. If this reaction occurs within a polymerase active site, the activation free energy value should be significantly lower because enzymes stabilize transition states and thereby reduce activation energy barriers [90]. Consistent with this expectation, the activation free energy for the rate-limiting concerted formation and cleavage of the P–O bonds (an S_N_2 reaction) within the active site of uncrosslinked Polβ is predicted to be 14 kcal/mol [41]. If the chemical step of DNA-crosslinked Polβ (Step 6, Fig. 8) has a similar activation free energy, this value would be ∼4 kcal/mol lower than our experimentally measured activation free energy (${\mathrm{\Delta }}{{G}^\ddagger }$) of 18.0 ± 0.3 kcal/mol (Fig. 7). This discrepancy indicates that the measured activation barrier reflects the dominant energetic contribution of Step 5, rather than the chemical step (Step 6) (Supplementary Table S3). This interpretation aligns with computational studies demonstrating that once a catalytically competent ensemble is achieved, the intrinsic phosphoryl-transfer barrier is comparatively modest [39–41, 91]. Collectively, our kinetic, thermodynamic, and structural results support a kinetic mechanism (Fig. 8) in which correct nucleotide incorporation by DNA-crosslinked Polβ is limited by a pre-chemical active-site reorganization (see below) that precedes phosphodiester bond formation (Supplementary Table S3).

### Structural features underlying the rate-limiting conformational change in DNA-crosslinked hPolβ

Having established that the rate-limiting step reflects localized pre-chemical reorganization (Step 5, Fig. 8), we next examined the structural features that are likely to underlie this conformational transition. Previously, our attempts to crystallize the ground-state ternary complex E‒DNA_n_●dNTP (Fig. 8) with crosslinked hPolβ were unsuccessful [7], a challenge shared by failed attempts with uncrosslinked polymerases due to inherent instability of the loosely bound ternary complex [20]. Consequently, the structure of E●DNA_n_●dNTP has generally been inferred to resemble that of E●DNA_n_ [20]. Accordingly, we compared two binary structures, (hPolβ‒DNA^dRP^)_A_ (Fig. 9A) and (hPolβ‒DNA^dRP^)_B_ (Fig. 9B), with the two ternary structures, (hPolβ‒DNA^dRP^●dNTP)_1_ (Fig. 9C) and (hPolβ‒DNA^dRP^●dNTP)_2_ (Fig. 9D), to reveal the structural basis of the rate-limiting Step 5 (Fig. 8). In both ternary structures, nucleotide binding induced a closed polymerase domain conformation, characterized by thumb subdomain closure (α-Helix N movement toward the nascent base pair and active site residue repositioning; Fig. 9A‒D), while the dRP lyase domain remained unchanged [7]. In addition to global thumb movement, structural and computational studies of uncrosslinked Polβ [33, 39–41, 82, 83, 91] indicate that conformational activation involves coordinated residue-level rearrangements, including repositioning of catalytic aspartates, rotation of active-site residues’ side chains, alignment and deprotonation of the primer 3′-OH, and progressive divalent metal coordination changes that together generate a reaction-competent configuration. Consistent with this broader view, (hPolβ‒DNA^dRP^●dNTP)_1_ and (hPolβ‒DNA^dRP^●dNTP)_2_ [7] capture ternary structures differing primarily in the upstream primer 3′-OH orientation relative to the dCTP Pα and the number of bound divalent metal ions (0.834 Å RMSD), features consistent with subtle pre-chemical active-site rearrangements corresponding to Step 5i within Step 5 (Fig. 8).

Additional evidence for the importance of this structural reorganization comes from femtosecond fluorescence studies of polymerase complexes, which show that water molecules at the substrate-binding interface exhibit restricted mobility and dynamic rearrangement upon ternary complex formation [79]. These observations highlight the role of solvent dynamics during assembly of the catalytically competent complex E′‒DNA_n_●dNTP (Fig. 8). DNA crosslinking increases the stability of the ternary complex relative to uncrosslinked hPolβ and likely slows the conformational transition required to reach the activated catalytic state. This effect is consistent with the observed ∼4-fold reduction in *k*_p_ for correct dCTP incorporation [7].

## Conclusions

In this study, we established a minimal kinetic mechanism for correct nucleotide incorporation by DNA-crosslinked hPolβ (Fig. 8) through integration of structural, kinetic, and thermodynamic analyses. Sulfur elemental effect measurements indicate that the phosphodiester bond-forming chemistry is not rate-limiting for correct nucleotide incorporation. Pulse-chase experiments reveal the formation of a kinetically competent ternary complex preceding the chemical step, while viscosity experiments exclude large-scale domain motions as the dominant kinetic barrier. Together with activation-energy analysis, these results demonstrate that correct nucleotide incorporation by DNA-crosslinked hPolβ is limited by a pre-chemical conformational reorganization that arranges divalent metal ions and water molecules, primer alignment, and active-site geometry into a reaction-competent state.

These findings demonstrate that the rate-limiting steps for correct and incorrect nucleotide incorporation by DNA-crosslinked hPolβ align with those determined for kinetically characterized uncrosslinked DNA polymerases, reverse transcriptases, and RNA polymerases, including uncrosslinked hPolβ (Supplementary Table S1). DNA crosslinking was found to enhance catalytic flux by stabilizing otherwise transient intermediates and reducing dissociative partitioning, a strategy that may extend to other DNA repair enzymes. We also determined key microscopic rate constants, including rate constants for various steps (*k*_2_, *k*_5_, and *k*_9_), the internal equilibrium constant (*k*_6_/*k*_-6_) for the chemical step, and the *K*_d_ (*k*_-4_/*k*_4_) for nucleotide binding (Fig. 8). Although additional analyses will be required to fully resolve all remaining microscopic rate constants in Fig. 8, the present study establishes the first minimal kinetic and thermodynamic framework for correct nucleotide incorporation by a DNA-crosslinked polymerase and demonstrates that conformational gating is an intrinsic and conserved feature of polymerase catalysis under physiologically relevant conditions.

## Supplementary Material

gkag539_Supplemental_File

## Data Availability

Atomic coordinates and structure factors for the reported crystal structure have been deposited in Protein Data Bank (PDB) with accession code 9Y62. The Protein Data Bank for figures generated from previously reported crystal structures are reported in the text. All other data are included in the manuscript and/or SI Appendix.

## References

[1] Carter RJ, Parsons JL. Base excision repair, a pathway regulated by posttranslational modifications. Mol Cell Biol. 2016;36:1426–37. 10.1128/MCB.00030-16. 26976642 PMC4859697

[2] Krokan HE, Bjoras M. Base excision repair. Cold Spring Harb Perspect Biol. 2013;5:a012583. 10.1101/cshperspect.a012583. 23545420 PMC3683898

[3] Lindahl T . DNA repair enzymes. Annu Rev Biochem. 1982;51:61–87. 10.1146/annurev.bi.51.070182.000425. 6287922

[4] Lindahl T, Andersson A. Rate of chain breakage at apurinic sites in double-stranded deoxyribonucleic acid. Biochemistry. 1972;11:3618–23. 10.1021/bi00769a019. 4559796

[5] Lindahl T, Nyberg B. Rate of depurination of native deoxyribonucleic acid. Biochemistry. 1972;11:3610–8. 10.1021/bi00769a018. 4626532

[6] Olinski R, Jurgowiak M, Zaremba T. Uracil in DNA—its biological significance. Mutation Res/Rev Mutation Res. 2010;705:239–45. 10.1016/j.mrrev.2010.08.001. 20709185

[7] Kumar A, Reed AJ, Zahurancik WJ et al. Interlocking activities of DNA polymerase β in the base excision repair pathway. Proc Natl Acad Sci USA. 2022;119:e2118940119. 10.1073/pnas.2118940119. 35238634 PMC8915974

[8] Sweasy JB, Julia ML, Eckert KA. DNA polymerases and human diseases. Radiat Res. 2006;166:693–714. 10.1667/RR0706.1. 17067213

[9] Qin J, Zhu Y, Ding Y et al. DNA polymerase β deficiency promotes the occurrence of esophageal precancerous lesions in mice. Neoplasia. 2021;23:663–75. 10.1016/j.neo.2021.05.001. 34144266 PMC8217306

[10] Mentegari E, Kissova M, Bavagnoli L et al. DNA polymerases λ and β: the double-edged swords of DNA repair. Genes. 2016;7:57. 10.3390/genes7090057. 27589807 PMC5042388

[11] Nemec AA, Murphy DL, Donigan KA et al. The S229L colon tumor-associated variant of DNA polymerase β induces cellular transformation as a result of decreased polymerization efficiency. J Biol Chem. 2014;289:13708–16. 10.1074/jbc.M114.550400. 24668809 PMC4022845

[12] Donigan KA, Sun K-W, Nemec AA et al. Human POLB gene is mutated in high percentage of colorectal tumors. J Biol Chem. 2012;287:23830–9. 10.1074/jbc.M111.324947. 22577134 PMC3390656

[13] Idriss HT, Al-Assar O, Wilson SH. DNA polymerase β. Int J Biochem Cell Biol. 2002;34:321–4. 10.1016/S1357-2725(01)00131-5. 11854030

[14] Huang J, Alnajjar KS, Mahmoud MM et al. The nature of the DNA substrate influences pre-catalytic conformational changes of DNA polymerase β. J Biol Chem. 2018;293:15084–94. 10.1074/jbc.RA118.004564. 30068550 PMC6166726

[15] Werneburg BG, Ahn J, Zhong X et al. DNA polymerase β: pre-steady-state kinetic analysis and roles of arginine-283 in catalysis and fidelity. Biochemistry. 1996;35:7041–50. 10.1021/bi9527202. 8679529

[16] Ahn J, Kraynov VS, Zhong X et al. DNA polymerase β: effects of gapped DNA substrates on dNTP specificity, fidelity, processivity and conformational changes. Biochem J. 1998;331:79–87. 10.1042/bj3310079. 9512464 PMC1219323

[17] Vande Berg BJ, Beard WA, Wilson SH. DNA structure and aspartate 276 influence nucleotide binding to human DNA polymerase β. J Biol Chem. 2001;276:3408–16. 10.1074/jbc.M002884200. 11024043

[18] Johnson KA . Conformational coupling in DNA polymerase fidelity. Annu Rev Biochem. 1993;62:685–713. 10.1146/annurev.bi.62.070193.003345. 7688945

[19] Beard WA, Wilson SH. Structure and mechanism of DNA polymerase β. Biochemistry. 2014;53:2768–80. 10.1021/bi500139h. 24717170 PMC4018062

[20] Raper AT, Reed AJ, Suo Z. Kinetic mechanism of DNA polymerases: contributions of conformational dynamics and a third divalent metal ion. Chem Rev. 2018;118:6000–25. 10.1021/acs.chemrev.7b00685. 29863852

[21] Joyce CM, Benkovic SJ. DNA polymerase fidelity: kinetics, structure, and checkpoints. Biochemistry. 2004;43:14317–24. 10.1021/bi048422z. 15533035

[22] Wu WJ, Yang W, Tsai MD. How DNA polymerases catalyse replication and repair with contrasting fidelity. Nat Rev Chem. 2017;1:0068. 10.1038/s41570-017-0068.

[23] Maxwell BA, Suo Z. Recent insight into the kinetic mechanisms and conformational dynamics of Y-Family DNA polymerases. Biochemistry. 2014;53:2804–14. 10.1021/bi5000405. 24716482 PMC4018064

[24] Betancourt D, Seay TW, Zalenski N et al. Pre-steady-state kinetic studies of nucleotide incorporation into a single-nucleotide gapped DNA substrate catalyzed by human DNA polymerase β. Biochemistry. 2025;64:1032–41. 10.1021/acs.biochem.4c00804. 39931791

[25] Schlick T, Arora K, Beard WA et al. Perspective: pre-chemistry conformational changes in DNA polymerase mechanisms. Theor Chem Acc. 2012;131:1287. 10.1007/s00214-012-1287-7. 23459563 PMC3583561

[26] Johnson KA . The kinetic and chemical mechanism of high-fidelity DNA polymerases. Biochim Biophys Acta. 2010;1804:1041–8. 10.1016/j.bbapap.2010.01.006. 20079883 PMC3047511

[27] Zhong X, Patel SS, Werneburg BG et al. DNA polymerase β: multiple conformational changes in the mechanism of catalysis. Biochemistry. 1997;36:11891–900. 10.1021/bi963181j. 9305982

[28] Liptak C, Mahmoud MM, Eckenroth BE et al. I260Q DNA polymerase β highlights precatalytic conformational rearrangements critical for fidelity. Nucleic Acids Res. 2018;46:10740–56. 30239932 10.1093/nar/gky825 PMC6237750

[29] Whitaker AM, Freudenthal BD. History of DNA polymerase β X-ray crystallography. DNA Repair. 2020;93:102928. 10.1016/j.dnarep.2020.102928. 33087265 PMC7586737

[30] Bakhtina M, Lee S, Wang Y et al. Use of viscogens, dNTPαS, and rhodium(III) as Probes in Stopped-Flow Experiments To Obtain New Evidence for the Mechanism of Catalysis by DNA Polymerase β. Biochemistry. 2005;44:5177–87. 10.1021/bi047664w. 15794655

[31] Dunlap CA, Tsai M-D. Use of 2-aminopurine and tryptophan fluorescence as probes in kinetic analyses of DNA polymerase β. Biochemistry. 2002;41:11226–35. 10.1021/bi025837g. 12220188

[32] Bakhtina M, Roettger MP, Tsai M-D. Contribution of the reverse rate of the conformational step to polymerase β fidelity. Biochemistry. 2009;48:3197–208. 10.1021/bi802119f. 19231836

[33] Arndt JW, Gong W, Zhong X et al. Insight into the catalytic mechanism of DNA polymerase β: structures of intermediate complexes. Biochemistry. 2001;40:5368–75. 10.1021/bi002176j. 11330999

[34] Sucato CA, Upton TG, Kashemirov BA et al. Modifying the β,γ leaving-group bridging oxygen alters nucleotide incorporation efficiency, fidelity, and the catalytic mechanism of DNA polymerase β. Biochemistry. 2007;46:461–71. 10.1021/bi061517b. 17209556

[35] Oertell K, Chamberlain BT, Wu Y et al. Transition state in DNA polymerase β catalysis: rate-limiting chemistry altered by base-pair configuration. Biochemistry. 2014;53:1842–8. 10.1021/bi500101z. 24580380 PMC3985788

[36] Towle-Weicksel JB, Dalal S, Sohl CD et al. Fluorescence resonance energy transfer studies of DNA polymerase β: the critical role of fingers domain movements and a novel non-covalent step during nucleotide selection. J Biol Chem. 2014;289:16541–50. 10.1074/jbc.M114.561878. 24764311 PMC4047420

[37] Fijen C, Mahmoud MM, Kronenberg M et al. Using single-molecule FRET to probe the nucleotide-dependent conformational landscape of polymerase β-DNA complexes. J Biol Chem. 2020;295:9012–20. 10.1074/jbc.RA120.013049. 32385112 PMC7335799

[38] Alnajjar KS, Wang K, Alvarado-Cruz I et al. Modifying the Basicity of the dNTP Leaving Group Modulates Precatalytic Conformational Changes of DNA Polymerase β. Biochemistry. 2024;63:1412–22. 10.1021/acs.biochem.4c00065. 38780930 PMC11155676

[39] Radhakrishnan R, Schlick T. Orchestration of cooperative events in DNA synthesis and repair mechanism unraveled by transition path sampling of DNA polymerase β closing. Proc Natl Acad Sci USA. 2004;101:5970–5. 10.1073/pnas.0308585101. 15069184 PMC395907

[40] Lin P, Pedersen LC, Batra VK et al. Energy analysis of chemistry for correct insertion by DNA polymerase β. Proc Natl Acad Sci USA. 2006;103:13294–9. 10.1073/pnas.0606006103. 16938895 PMC1569157

[41] Klvaňa M, Bren U, Florián J. Uniform free-energy profiles of the P–O bond formation and cleavage reactions catalyzed by DNA polymerases β and λ. J Phys Chem B. 2016;120:13017–30. 27992186 10.1021/acs.jpcb.6b08581 PMC5217713

[42] Yamtich J, Sweasy JB. DNA polymerase family X: function, structure, and cellular roles. Biochim Biophys Acta. 2010;1804:1136–50. 10.1016/j.bbapap.2009.07.008. 19631767 PMC2846199

[43] Wang LK, Shuman S. Domain structure and mutational analysis of T4 polynucleotide kinase. J Biol Chem. 2001;276:26868–74. 10.1074/jbc.M103663200. 11335730

[44] Brown JA, Duym WW, Fowler JD et al. Single-turnover kinetic analysis of the mutagenic potential of 8-oxo-7,8-dihydro-2'-deoxyguanosine during gap-filling synthesis catalyzed by human DNA polymerases λ and β. J Mol Biol. 2007;367:1258–69. 10.1016/j.jmb.2007.01.069. 17321545

[45] Brown JA, Fowler JD, Suo Z. Kinetic basis of nucleotide selection employed by a protein template-dependent DNA polymerase. Biochemistry. 2010;49:5504–10. 10.1021/bi100433x. 20518555 PMC2907478

[46] Johnson KA . In: Sigman DS (ed.), The Enzymes. San Diego: Academic Press, Vol. 20, 1992, pp. 1–61. 10.1016/S1874-6047(08)60019-0.

[47] Johnson KA . Rapid quench kinetic analysis of polymerases, adenosinetriphosphatases, and enzyme intermediates. Methods Enzymol. 1995;249:38–61. 7791620 10.1016/0076-6879(95)49030-2

[48] Zhang L, Brown JA, Newmister SA et al. Polymerization fidelity of a replicative DNA polymerase from the hyperthermophilic archaeon Sulfolobus solfataricus P2. Biochemistry. 2009;48:7492–501. 10.1021/bi900532w. 19456141

[49] Sherrer SM, Brown JA, Pack LR et al. Mechanistic studies of the bypass of a bulky single-base lesion catalyzed by a Y-family DNA polymerase. J Biol Chem. 2009;284:6379–88. 10.1074/jbc.M808161200. 19124465 PMC2649090

[50] Fowler JD, Brown JA, Johnson KA et al. Kinetic investigation of the inhibitory effect of gemcitabine on DNA polymerization catalyzed by human mitochondrial DNA polymerase. J Biol Chem. 2008;283:15339–48. 10.1074/jbc.M800310200. 18378680 PMC2397453

[51] Brown JA, Newmister SA, Fiala KA et al. Mechanism of double-base lesion bypass catalyzed by a Y-family DNA polymerase. Nucleic Acids Res. 2008;36:3867–78. 10.1093/nar/gkn309. 18499711 PMC2475632

[52] Fiala KA, Suo Z. Mechanism of DNA polymerization catalyzed by *Sulfolobus solfataricus* P2 DNA polymerase IV. Biochemistry. 2004;43:2116–25. 10.1021/bi035746z. 14967051

[53] Brown JA, Suo Z. Elucidating the kinetic mechanism of DNA polymerization catalyzed by *Sulfolobus solfataricus* P2 DNA polymerase B1. Biochemistry. 2009;48:7502–11. 10.1021/bi9005336. 19456143

[54] Liu J, Tsai M-D. DNA polymerase β: pre-steady-state kinetic analyses of dATPαS stereoselectivity and alteration of the stereoselectivity by various metal ions and by site-directed mutagenesis. Biochemistry. 2001;40:9014–22. 10.1021/bi010646j. 11467964

[55] Kabsch W . XDS. Acta Crystallogr D Biol Crystallogr. 2010;66:125–32. 10.1107/S0907444909047337. 20124692 PMC2815665

[56] Evans PR, Murshudov GN. How good are my data and what is the resolution?. Acta Crystallogr D Biol Crystallogr. 2013;69:1204–14. 10.1107/S0907444913000061. 23793146 PMC3689523

[57] McCoy AJ, Grosse-Kunstleve RW, Adams PD et al. Phaser crystallographic software. J Appl Crystallogr. 2007;40:658–74. 10.1107/S0021889807021206. 19461840 PMC2483472

[58] Emsley P, Cowtan K. Coot: model-building tools for molecular graphics. Acta Crystallogr D Biol Crystallogr. 2004;60:2126–32. 10.1107/S0907444904019158. 15572765

[59] Afonine PV, Grosse-Kunstleve RW, Echols N et al. Towards automated crystallographic structure refinement with phenix.refine. Acta Crystallogr D Biol Crystallogr. 2012;68:352–67. 10.1107/S0907444912001308. 22505256 PMC3322595

[60] Herschlag D, Piccirilli JA, Cech TR. Ribozyme-catalyzed and nonenzymatic reactions of phosphate diesters: rate effects upon substitution of sulfur for a nonbridging phosphoryl oxygen atom. Biochemistry. 1991;30:4844–54. 10.1021/bi00234a003. 2036355

[61] Perera L, Freudenthal BD, Beard WA et al. Revealing the role of the product metal in DNA polymerase β catalysis. Nucleic Acids Res. 2017;45:2736–45. 28108654 10.1093/nar/gkw1363 PMC5389463

[62] Zahurancik WJ, Klein SJ, Suo Z. Kinetic mechanism of DNA polymerization catalyzed by human DNA polymerase ε. Biochemistry. 2013;52:7041–9. 10.1021/bi400803v. 24020356 PMC3859840

[63] Brown JA, Pack LR, Sanman LE et al. Efficiency and fidelity of human DNA polymerases λ and β during gap-filling DNA synthesis. DNA Repair. 2011;10:24–33. 10.1016/j.dnarep.2010.09.005. 20961817 PMC3065367

[64] Brown JA, Pack LR, Fowler JD et al. Presteady state kinetic investigation of the incorporation of anti-hepatitis B nucleotide analogues catalyzed by noncanonical human DNA polymerases. Chem Res Toxicol. 2012;25:225–33. 10.1021/tx200458s. 22132702 PMC3259259

[65] García-Díaz M, Bebenek K, Kunkel TA et al. Identification of an intrinsic 5^′^-deoxyribose-5-phosphate lyase activity in human DNA polymerase λ: a possible role in base excision repair. J Biol Chem. 2001;276:34659–63. 11457865 10.1074/jbc.M106336200

[66] Longley MJ, Prasad R, Srivastava DK et al. Identification of 5^′^-deoxyribose phosphate lyase activity in human DNA polymerase γ and its role in mitochondrial base excision repair *in vitro*. Proc Natl Acad Sci USA. 1998;95:12244–8. 10.1073/pnas.95.21.12244. 9770471 PMC22816

[67] Bebenek K, Tissier A, Frank EG et al. 5^′^-Deoxyribose phosphate lyase activity of human DNA polymerase ι *in vitro*. Science. 2001;291:2156–9. 10.1126/science.1058386. 11251121

[68] Miropolskaya N, Petushkov I, Kulbachinskiy A et al. Identification of amino acid residues involved in the dRP-lyase activity of human Pol ι. Sci Rep. 2017;7:10194. 10.1038/s41598-017-10668-5. 28860552 PMC5579206

[69] Prasad R, Longley MJ, Sharief FS et al. Human DNA polymerase θ possesses 5′-dRP lyase activity and functions in single-nucleotide base excision repair *in vitro*. Nucleic Acids Res. 2009;37:1868–77. 10.1093/nar/gkp035. 19188258 PMC2665223

[70] Prasad R, Poltoratsky V, Hou EW et al. Rev1 is a base excision repair enzyme with 5΄-deoxyribose phosphate lyase activity. Nucleic Acids Res. 2016;44:10824–33. 10.1093/nar/gkw869. 27683219 PMC5159550

[71] Fiala KA, Abdel-Gawad W, Suo Z. Pre-steady-state kinetic studies of the fidelity and mechanism of polymerization catalyzed by truncated human DNA polymerase λ. Biochemistry. 2004;43:6751–62. 10.1021/bi049975c. 15157109

[72] Case BC, Scoccia L, Zhao Z et al. DNA polymerase lambda autoinhibition is relieved via Ku interaction during non-homologous end joining. Nucleic Acids Res. 2026;54:gkag114. 10.1093/nar/gkag114. 41700088 PMC12910108

[73] Kati WM, Johnson KA, Jerva LF et al. Mechanism and fidelity of HIV reverse transcriptase. J Biol Chem. 1992;267:25988–97. 10.1016/S0021-9258(18)35706-5. 1281479

[74] Fiala KA, Sherrer SM, Brown JA et al. Mechanistic consequences of temperature on DNA polymerization catalyzed by a Y-family DNA polymerase. Nucleic Acids Res. 2008;36:1990–2001. 10.1093/nar/gkn004. 18276639 PMC2346602

[75] Patel SS, Wong I, Johnson KA. Pre-steady-state kinetic analysis of processive DNA replication including complete characterization of an exonuclease-deficient mutant. Biochemistry. 1991;30:511–25. 10.1021/bi00216a029. 1846298

[76] Tsai YC, Johnson KA. A new paradigm for DNA polymerase specificity. Biochemistry. 2006;45:9675–87. 10.1021/bi060993z. 16893169 PMC7526746

[77] Lee HR, Wang M, Konigsberg W. The reopening rate of the fingers domain is a determinant of base selectivity for RB69 DNA polymerase. Biochemistry. 2009;48:2087–98. 10.1021/bi8016284. 19228036 PMC2845987

[78] Graves SW, Johnson AA, Johnson KA. Expression, purification, and initial kinetic characterization of the large subunit of the human mitochondrial DNA polymerase. Biochemistry. 1998;37:6050–8. 10.1021/bi972685u. 9558343

[79] Qin Y, Yang Y, Zhang L et al. Direct probing of solvent accessibility and mobility at the binding interface of polymerase (Dpo4)-DNA complex. J Phys Chem A. 2013;117:13926–34. 10.1021/jp410051w. 24308461 PMC3903812

[80] Gregory MT, Gao Y, Cui Q et al. Multiple deprotonation paths of the nucleophile 3^′^-OH in the DNA synthesis reaction. Proc Natl Acad Sci USA. 2021;118:e2103990118. 10.1073/pnas.2103990118. 34088846 PMC8201771

[81] Reed AJ, Vyas R, Raper AT et al. Structural Insights into the post-chemistry steps of nucleotide incorporation catalyzed by a DNA polymerase. J Am Chem Soc. 2017;139:465–71. 10.1021/jacs.6b11258. 27959534 PMC6634981

[82] Vyas R, Reed AJ, Tokarsky EJ et al. Viewing human DNA polymerase β faithfully and unfaithfully bypass an oxidative lesion by time-dependent crystallography. J Am Chem Soc. 2015;137:5225–30. 10.1021/jacs.5b02109. 25825995 PMC4519080

[83] Freudenthal BD, Beard WA, Shock DD et al. Observing a DNA polymerase choose right from wrong. Cell. 2013;154:157–68. 10.1016/j.cell.2013.05.048. 23827680 PMC3924593

[84] Nakamura T, Zhao Y, Yamagata Y et al. Watching DNA polymerase η make a phosphodiester bond. Nature. 2012;487:196–201. 10.1038/nature11181. 22785315 PMC3397672

[85] Gao Y, Yang W. Capture of a third Mg^2+^ is essential for catalyzing DNA synthesis. Science. 2016;352:1334–7. 10.1126/science.aad9633. 27284197 PMC6320252

[86] Jamsen JA, Beard WA, Pedersen LC et al. Time-lapse crystallography snapshots of a double-strand break repair polymerase in action. Nat Commun. 2017;8:253. 10.1038/s41467-017-00271-7. 28811466 PMC5557891

[87] Beese LS, Steitz TA. Structural basis for the 3^′^-5^′^ exonuclease activity of *Escherichia coli* DNA polymerase I: a two metal ion mechanism. EMBO J. 1991;10:25–33. 10.1002/j.1460-2075.1991.tb07917.x. 1989886 PMC452607

[88] Capson TL, Peliska JA, Kaboord BF et al. Kinetic characterization of the polymerase and exonuclease activities of the gene 43 protein of bacteriophage T4. Biochemistry. 1992;31:10984–94. 10.1021/bi00160a007. 1332748

[89] Florián J, Goodman MF, Warshel A. Computer simulation of the chemical catalysis of dna polymerases: discriminating between alternative nucleotide insertion mechanisms for T7 DNA polymerase. J Am Chem Soc. 2003;125:8163–77. 10.1021/ja028997o. 12837086

[90] Pauling L . Molecular architecture and biological reactions. Chem Eng News Archive. 1946;24:1375–7. 10.1021/cen-v024n010.p1375.

[91] Abashkin YG, Erickson JW, Burt SK. Quantum chemical investigation of enzymatic activity in DNA polymerase β. A mechanistic study. J Phys Chem B. 2001;105:287–92. 10.1021/jp003629x.

