# Supplementary material for: Conformational gating governs nucleotide incorporation by a DNA-crosslinked polymerase
Source: Nucleic Acids Res. 2026 Jun 2;54(10):gkag539. doi: 10.1093/nar/gkag539 (PMC13227109; doi:10.1093/nar/gkag539)
Supplement: gkag539_Supplemental_File [file gkag539_supplemental_file.pdf]

Supplemental Information

Conformational gating governs nucleotide incorporation by a DNA-crosslinked polymerase

Daniel Betancourt, Amit Gaur, Turner W. Seay, Nikita Zalenski, Zucai Suo*

Department of Biomedical Sciences, College of Medicine, Florida State University, Tallahassee, FL 32306, USA

*To whom correspondence should be addressed: Zucai Suo, Department of Biomedical Sciences, College of Medicine, Florida State University, Tallahassee, FL 32306, USA; Tel.: (850) 645-2501; E-mail: zucai.suo@med.fsu.edu

Figures S1-S5

Tables S1-S3

References 1-32

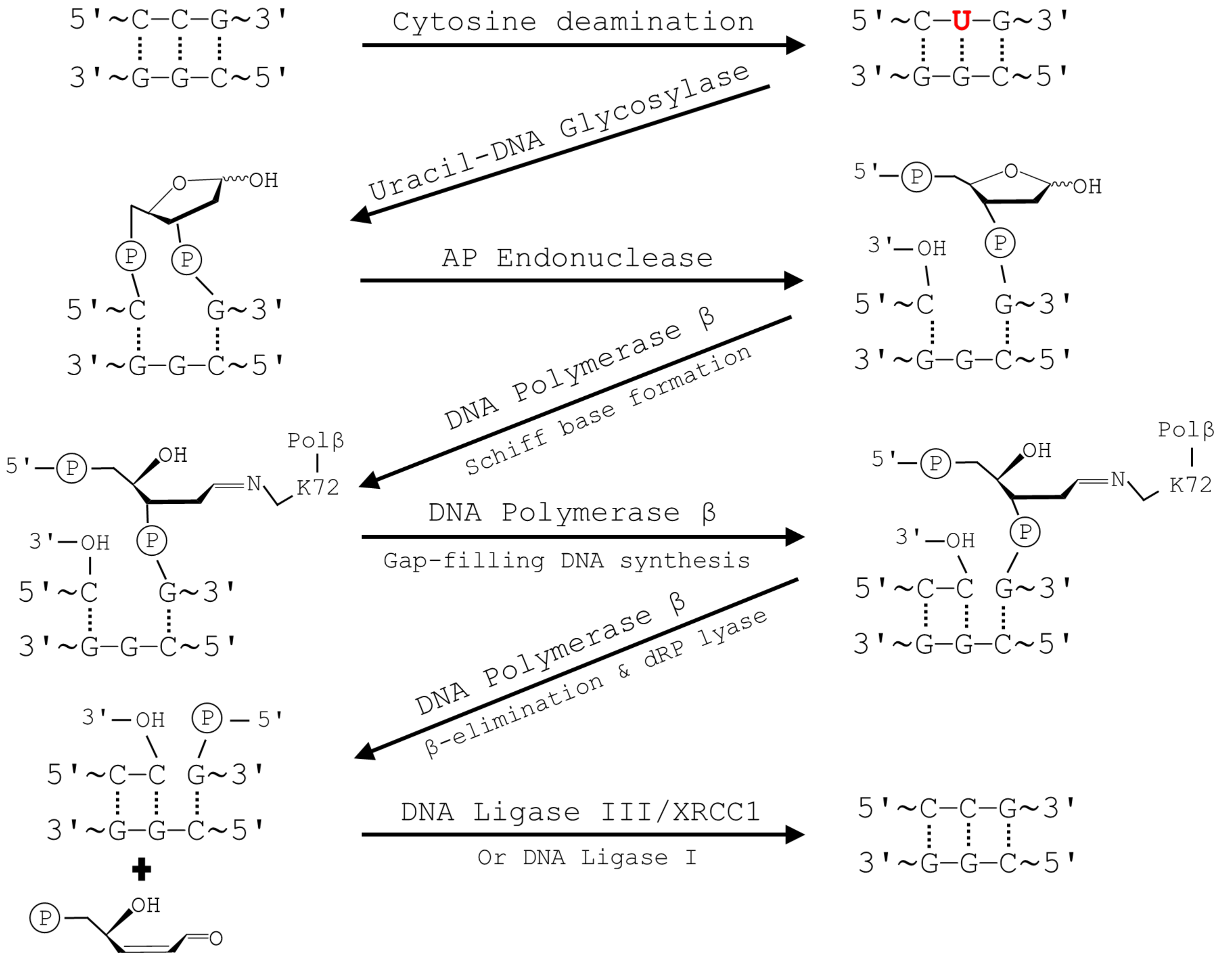

Figure S1. Recently revised base excision repair pathway (1). The deoxyuridine (dU) is shown in red. A phosphate moiety is depicted as a circled "P" (℗).

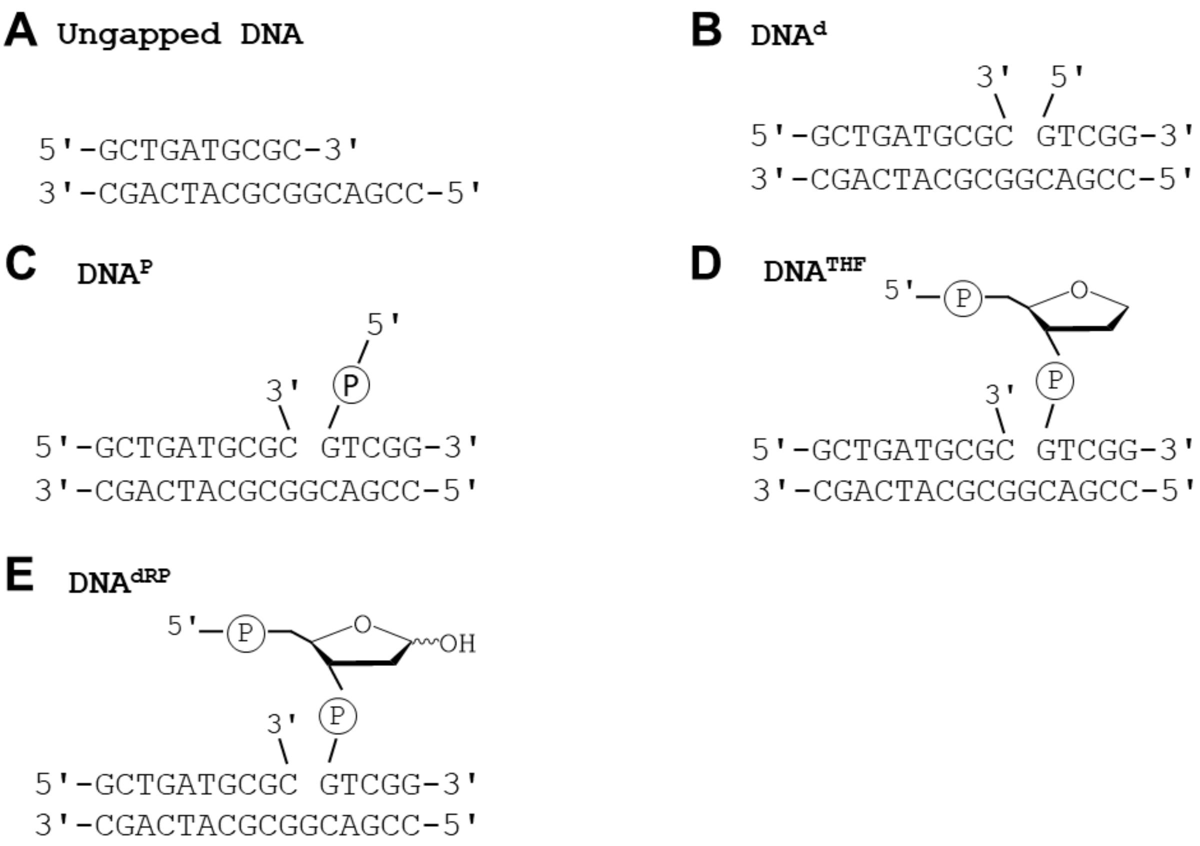

Figure S2. Examples of DNA substrates used in published mechanistic studies of Polβ (2-4). The DNA oligomers' sequences and lengths can differ from those shown in the panels adapted from the literature. These include an ungapped substrate (A) and gapped substrates, with gaps ranging from one to a few nucleotides (B-D; single gapped substrates shown). A physiologically relevant substrate containing a dRP moiety is shown in (E). A phosphate group is depicted as a circled "P" (℗).

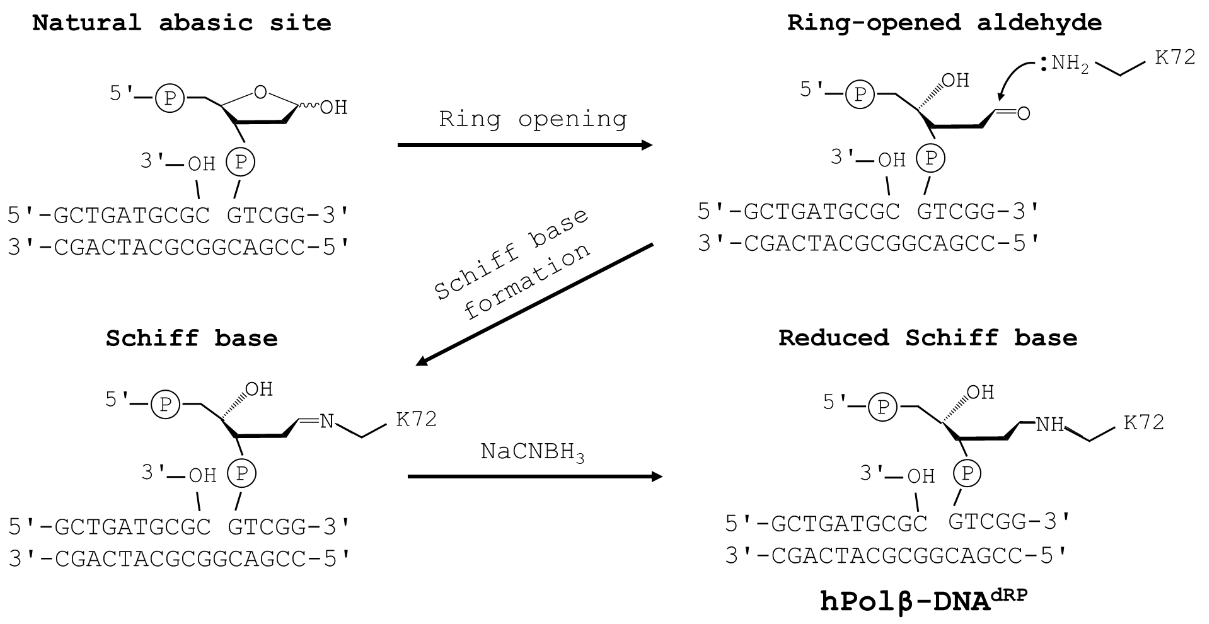

Figure S3. Chemical mechanism for crosslinking of hPolβ to DNA^{dRP}. A phosphate moiety is depicted as a circled "P" (℗).

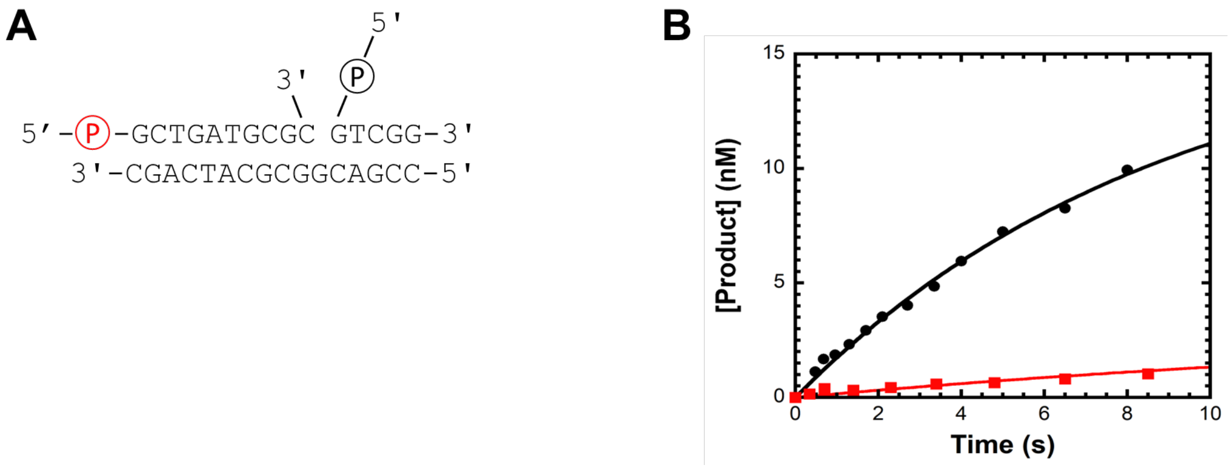

Figure S4. Sulfur elemental effect on correct nucleotide incorporation by uncrosslinked hPolβ in the presence of an equimolar amount of DNA. **(A)** The double-stranded DNA^P substrate used for this assay. The upstream 10mer primer was [^{32}P]-labeled (Ⓟ) while the downstream 5mer was 5′-phosphorylated but not radiolabeled (Ⓟ). (B) A preincubated solution of uncrosslinked hPolβ (30 nM) and 5′-^{32}P-labeled DNA^P (30 nM) was rapidly mixed with 25 µM dCTP (●) or *Sp*-dCTPαS (■) for various times before being quenched. The time courses were fitted to Equation 1 (see Materials and Methods), yielding a reaction amplitude of 16 ± 2 nM and a k_{obs} of 0.11 ± 0.02 s^{-1} for dCTP incorporation, and 3.5 ± 0.1 nM and 0.048 ± 0.003 s^{-1} for *Sp*-dCTPαS incorporation.

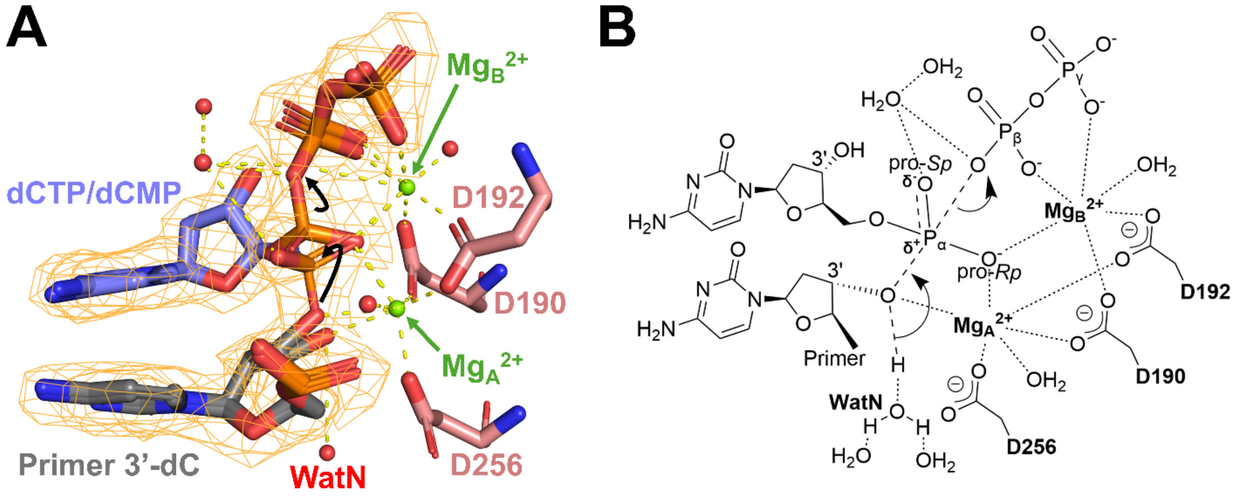

Figure S5. Proposed transition state based on a reaction-state crystal structure of uncrosslinked hPolβ. **(A)** Zoomed reaction-state structure (PDB: 4KLE). Following 10 s of Ca^{2+} to Mg^{2+} exchange *in crystallo*, an electron density *2Fo–Fc* omit map (light orange; 1.5σ) displays the upstream primer 3′-dC, 70% remaining dCTP, and 30% incorporated dCMP. The concurrent phosphodiester bond formation and Pα-O bond breakage, accompanied by the steric inversion of the Pα geometry, provide direct evidence for an S_N2 reaction between the primer 3′-dC and dCTP. Mg^{2+} (green) and water (red) are shown as spheres. **(B)** Proposed transition state for phosphodiester bond formation catalyzed by uncrosslinked hPolβ as derived from the reaction-state structure in (A). Dashed lines represent bonds undergoing cleavage and formation in the S_N2 reaction, while dotted lines denote either hydrogen bonds or the coordination bonds to the Mg^{2+} ions.

Table S1. Rate-limiting step of uncrosslinked DNA polymerases, reverse transcriptases, and RNA polymerases as determined by sulfur elemental effect and/or pulse-chase/pulse-quench kinetic assays. Cited references are in parentheses.

	Correct dNTP				Incorrect dNTP	
**Polymerase[a]**	**Sulfur elemental effect[b]**	**Rate limiting Step**	**Pulse-chase/pulse-quench[c]**	**Rate limiting Step**	**Sulfur elemental effect[b]**	**Rate limiting Step**
Klenow fragment	1.6, ~1 (5,6)	Step 3[f]	>1 (6,7)	Step 3	12.5[d]-65[e] (8,9)	Step 4[f]
Geobacillus ana-tolicus fragment	2.7 (10)	Step 3	ND[g]	ND	15 (10)	Step 4
T7 DNA Pol[d]	3.1 (11)	Step 3	>1 (11)	Step 3	19-34 (12)	Step 4
HIV-1 RT	1.2, 1.3 (13-15)	Step 3	>1 (13)	Step 3	18[h] (16)	Step 4
DNA Polβ	4.3 (17), 2.1 (18), 4.8-6.6 (19), 7 (20), 5.0 (21), 4.0 (22)	Step 3	>1 (22)	Step 3	9 (18), 5.6-12 (19), 64.6 (22)	Step 4
Dpo4	1.4 (23)	Step 3	>1 (23)	Step 3	6 (23)	Step 4
Dbh	9 (24)	Step 3	ND	ND	AL[i]	Step 4
DNA PolB1	1.5 (25)	Step 3	>1 (25)	ND	36 (25)	Step 4
DNA Polε[d]	0.9, 1.1 (26,27)	Step 3	>1 (26)	Step 3	167, 46 (26,27)	Step 4
RNA Pol II	ND	ND	>1 (28)	Step 3	ND	ND
T7 RNA Pol	0.7 (29)	Step 3	=1 (29)	Step 3	ND	ND
Poliovirus RNA-dependent RNA Pol	3.8-4.2 (30)	Step 3	>1 (30,31)	Step 3	ND	ND
Human mito-chondria RNA Pol	ND	ND	>1 (32)	Step 3	ND	ND

[a]Pol and RT denote polymerase and reverse transcriptase, respectively.
[b]Ratio of the observed nucleotide incorporation rate constants $k_{obs,\ dNTP}/k_{obs,\ Sp\text{-}dNTP\alpha S}$
[c]Ratio of the reaction amplitudes $A_{pulse\text{-}chase}/A_{pulse\text{-}quench}$
[d]3′→5′ exonuclease-deficient form of the polymerase
[e]3′→5′ exonuclease-proficient form of the polymerase
[f]Step 3 and Step 4 in Figure 2A.
[g]ND means not determined.
[h]incorporation on top of mismatch
[i]AL means above the limit of detection of the assay

Table S2. X-ray diffraction data collection and refinement statistics for the hPolβ–DNA^{dRP}●*Sp*-dCTPαS ternary complex.

**Data collection***	
**Wavelength (Å)**	0.979
**Space group**	P 1 2_1 1
**Cell dimensions (Å)**	a=47.68, b=77.88, c=54.78,
**Cell dimensions (°)**	α=90.00, β=105.63, γ=90.00
**Resolution range (Å)**	45.92 - 2.10 (2.16-2.10)
R_{merge}	0.09 (3.24)
R_{meas}	0.11 (3.84)
I/σI	9.60 (0.40)
**Completeness (%)**	90.88 (72.70)
**Redundancy**	3.8 (3.40)
**Total reflections**	77326 (4586)
**Refinement**	
**Resolution (Å)**	45.92 - 2.10
**No. of Reflections**	20466
R_{work} / R_{free}‡	0.2010/0.2580
**No. Macromolecules**	3097
**No. Ligands**	18
**No. Solvent**	28
**B-factor for macromolecules (Å^2)**	60.0
**B-factor for ligands (Å^2)**	53.2 SDP / 86.3 QPJ / 85.7 CA / 58.4 NA
**B-factor for solvent (Å^2)**	53.7
**FSC average**	0.9362
**RMS bond lengths (Å)**	0.009
**RMS angles (°)**	1.85
**Ramachandran favored (%)**	96.21
**Ramachandran allowed (%)**	3.15
**Ramachandran outliers (%)**	0.63
**Clash score**	6.92
**Rotamer outliers (%)**	3.24

* Highest resolution shell is shown in parenthesis.

‡ R value = $\Sigma||F_o| - |F_c||/\Sigma|F_o|$, where F_o and F_c are observed and calculated structure factor amplitudes, respectively

Table S3. Summary of kinetic parameters for correct dNTP incorporation by DNA-crosslinked hPolβ (Figure 8) and evidence for the rate-limiting step.

Elementary Step	Parameter	Value	Tempera-ture	Notes
Step 1	k_{-1}	~0.93 s^{-1}	25 °C	Estimated from uncrosslinked hPolβ using DNA^{P} substrate (22)
Step 2	k_2	4.5 s^{-1}	25 °C	Rapid Schiff base formation (1)
Step 4	K_d (k_{-4}/k_4)	0.38 μM	25 °C	~70-fold decrease relative to uncrosslinked hPolβ (1)
Step 5	k_5	0.72 s^{-1}	25 °C	~4-fold lower value than uncrosslinked hPolβ (~20-fold higher in k_p/K_d) (1)
Step 6	k_6/k_{-6}	~2	37 °C	~2-fold increase in forward flux relative to un-crosslinked hPolβ (22)
Step 9	k_9	~0.14 s^{-1}	25 °C	The rate of disappearance of the β-elimination product generated by the dRP lyase activity (1)
Step 10	k_{10}	~0.93 s^{-1}	37 °C	Estimated from uncrosslinked hPolβ using DNA^{P} substrate (22)
Pulse-quench /Pulse-chase	$A_{pulse\text{-}chase}$ $/A_{pulse\text{-}quench}$	1.5 ± 0.1	37 °C	The ratio indicates the existence of E′–DNA_n•dNTP, and that Step 5 is rate-limiting.
Solvent viscosity dependence	k_{obs} ($\cong k_5$)	~0.72 s^{-1}	37 °C	No viscosity dependence of k_{obs} indicates that large-scale domain motions are not rate-limit-ing, and that Step 5i is rate-limiting.
Sulfur elemental effect	$k_{obs,dCTP}$ / $k_{obs,Sp\text{-}dCTP\alpha S}$	3.7 ± 0.4	37 °C	The small sulfur elemental effect suggests that the chemical step (k_6) is not rate limiting.
Eyring analysis	$\Delta G^{\ddagger}$	18 kcal/mol	37 °C	Exceeds 14 kcal/mol (computed activation free energy for the rate-limiting chemical step); the chemical step (k_6) is not rate limiting.

References

1. Kumar, A., Reed, A.J., Zahurancik, W.J. *et al.* (2022) Interlocking activities of DNA polymerase beta in the base excision repair pathway. *Proc Natl Acad Sci U S A*, **119**, e2118940119.

2. Raper, A.T., Reed, A.J. and Suo, Z. (2018) Kinetic Mechanism of DNA Polymerases: Contributions of Conformational Dynamics and a Third Divalent Metal Ion. *Chem Rev*, **118**, 6000–6025.

3. Joyce, C.M. and Benkovic, S.J. (2004) DNA polymerase fidelity: kinetics, structure, and checkpoints. *Biochemistry*, **43**, 14317–14324.

4. Wu, W.J., Yang, W. and Tsai, M.D. (2017) How DNA polymerases catalyse replication and repair with contrasting fidelity. *Nat. Rev. Chem.* , **1**, 0068.

5. Mizrahi, V., Henrie, R.N., Marlier, J.F. *et al.* (1985) Rate-limiting steps in the DNA polymerase I reaction pathway. *Biochemistry*, **24**, 4010–4018.

6. Prakasha Gowda, A.S. and Spratt, T.E. (2017) Active Site Interactions Impact Phosphoryl Transfer during Replication of Damaged and Undamaged DNA by Escherichia coli DNA Polymerase I. *Chemical Research in Toxicology*, **30**, 2033–2043.

7. Dahlberg, M.E. and Benkovic, S.J. (1991) Kinetic mechanism of DNA polymerase I (Klenow fragment): identification of a second conformational change and evaluation of the internal equilibrium constant. *Biochemistry*, **30**, 4835–4843.

8. Eger, B.T. and Benkovic, S.J. (1992) Minimal kinetic mechanism for misincorporation by DNA polymerase I (Klenow fragment). *Biochemistry*, **31**, 9227–9236.

9. Kuchta, R.D., Benkovic, P. and Benkovic, S.J. (1988) Kinetic mechanism whereby DNA polymerase I (Klenow) replicates DNA with high fidelity. *Biochemistry*, **27**, 6716–6725.

10. Çağlayan, M. and Bilgin, N.e. (2012) Temperature dependence of accuracy of DNA polymerase I from Geobacillus anatolicus. *Biochimie*, **94**, 1968–1973.

11. Patel, S.S., Wong, I. and Johnson, K.A. (1991) Pre-steady-state kinetic analysis of processive DNA replication including complete characterization of an exonuclease-deficient mutant. *Biochemistry*, **30**, 511–525.

12. Wong, I., Patel, S.S. and Johnson, K.A. (1991) An induced-fit kinetic mechanism for DNA replication fidelity: direct measurement by single-turnover kinetics. *Biochemistry*, **30**, 526–537.

13. Hsieh, J.C., Zinnen, S. and Modrich, P. (1993) Kinetic mechanism of the DNA-dependent DNA polymerase activity of human immunodeficiency virus reverse transcriptase. *J Biol Chem*, **268**, 24607–24613.

14. Radzio, J. and Sluis-Cremer, N. (2005) Stereo-selectivity of HIV-1 reverse transcriptase toward isomers of thymidine-5'-O-1-thiotriphosphate. *Protein Sci*, **14**, 1929–1933.

15. Ray, A.S., Murakami, E., Basavapathruni, A. *et al.* (2003) Probing the Molecular Mechanisms of AZT Drug Resistance Mediated by HIV-1 Reverse Transcriptase Using a Transient Kinetic Analysis. *Biochemistry*, **42**, 8831–8841.

16. Zinnen, S., Hsieh, J.C. and Modrich, P. (1994) Misincorporation and mispaired primer extension by human immunodeficiency virus reverse transcriptase. *J Biol Chem*, **269**, 24195–24202.

17. Werneburg, B.G., Ahn, J., Zhong, X. *et al.* (1996) DNA Polymerase β: Pre-Steady-State Kinetic Analysis and Roles of Arginine-283 in Catalysis and Fidelity. *Biochemistry*, **35**, 7041–7050.

18. Vande Berg, B.J., Beard, W.A. and Wilson, S.H. (2001) DNA Structure and Aspartate 276 Influence Nucleotide Binding to Human DNA Polymerase β. *Journal of Biological Chemistry*, **276**, 3408–3416.

19. Ahn, J., Kraynov, V.S., Zhong, X. *et al.* (1998) DNA polymerase β: effects of gapped DNA substrates on dNTP specificity, fidelity, processivity and conformational changes. *Biochemical Journal*, **331**, 79–87.

20. Bakhtina, M., Lee, S., Wang, Y. *et al.* (2005) Use of Viscogens, dNTPαS, and Rhodium(III) as Probes in Stopped-Flow Experiments To Obtain New Evidence for the Mechanism of Catalysis by DNA Polymerase β. *Biochemistry*, **44**, 5177–5187.

21. Liu, J. and Tsai, M.-D. (2001) DNA Polymerase β: Pre-Steady-State Kinetic Analyses of dATPαS Stereoselectivity and Alteration of the Stereoselectivity by Various Metal Ions and by Site-Directed Mutagenesis. *Biochemistry*, **40**, 9014–9022.

22. Betancourt, D., Seay, T.W., Zalenski, N. *et al.* (2025) Pre-steady-state kinetic studies of nucleotide incorporation into a single-nucleotide gapped DNA substrate catalyzed by human DNA polymerase β. *Biochemistry*, **64**, 1032–1041.

23. Fiala, K.A. and Suo, Z. (2004) Mechanism of DNA Polymerization Catalyzed by *Sulfolobus solfataricus* P2 DNA Polymerase IV. *Biochemistry*, **43**, 2116–2125.

24. Cramer, J. and Restle, T. (2005) Pre-steady-state Kinetic Characterization of the DinB Homologue DNA Polymerase of Sulfolobus solfataricus. *Journal of Biological Chemistry*, **280**, 40552–40558.

25. Brown, J.A. and Suo, Z. (2009) Elucidating the Kinetic Mechanism of DNA Polymerization Catalyzed by *Sulfolobus solfataricus* P2 DNA Polymerase B1. *Biochemistry*, **48**, 7502–7511.

26. Zahurancik, W.J., Klein, S.J. and Suo, Z. (2013) Kinetic mechanism of DNA polymerization catalyzed by human DNA polymerase ε. *Biochemistry*, **52**, 7041–7049.

27. Zahurancik, W.J. and Suo, Z. (2020) Kinetic investigation of the polymerase and exonuclease activities of human DNA polymerase ε holoenzyme. *Journal of Biological Chemistry*, **295**, 17251–17264.

28. Kireeva, M.L., Nedialkov, Y.A., Cremona, G.H. *et al.* (2008) Transient Reversal of RNA Polymerase II Active Site Closing Controls Fidelity of Transcription Elongation. *Molecular Cell*, **30**, 557–566.

29. Anand, V.S. and Patel, S.S. (2006) Transient State Kinetics of Transcription Elongation by T7 RNA Polymerase. *Journal of Biological Chemistry*, **281**, 35677–35685.

30. Arnold, J.J. and Cameron, C.E. (2004) Poliovirus RNA-dependent RNA polymerase (3Dpol): pre-steady-state kinetic analysis of ribonucleotide incorporation in the presence of Mg2+. *Biochemistry*, **43**, 5126–5137.

31. Moustafa, I.M., Korboukh, V.K., Arnold, J.J. *et al.* (2014) Structural Dynamics as a Contributor to Error-prone Replication by an RNA-dependent RNA Polymerase. *Journal of Biological Chemistry*, **289**, 36229–36248.

32. Smidansky, E.D., Arnold, J.J., Reynolds, S.L. *et al.* (2011) Human Mitochondrial RNA Polymerase: Evaluation of the Single-Nucleotide-Addition Cycle on Synthetic RNA/DNA Scaffolds. *Biochemistry*, **50**, 5016–5032.
